# Specialized metabolites present in *Camellia reticulata* nectar inhibit the growth of nectar‐inhabiting microorganisms

**DOI:** 10.3389/fpls.2025.1557228

**Published:** 2025-03-04

**Authors:** Lijie Xun, Rong Huang, Qiongyan Li, Qingxin Meng, Rui Su, Xiaoman Wu, Renbin Zhang, Linshu Li, Xueyang Gong, Kun Dong

**Affiliations:** ^1^ Yunnan Provincial Engineering and Research Center for Sustainable Utilization of Honey Bee Resources, Eastern Bee Research Institute, College of Animal Science and Technology, Yunnan Agricultural University, Kunming, China; ^2^ Institute of Sericulture and Apiculture, Yunnan Academy of Agricultural Sciences, Mengzi, China; ^3^ Shaba State-owned Forest Farm of Tengchong, Forestry and Grassland Bureau of Tengchong, Tengchong, China; ^4^ Animal Husbandry Workstation of Tengchong, Agriculture and Rural Affairs Bureau of Tengchong, Tengchong, China

**Keywords:** *Camellia reticulata*, nectar, nectar microorganisms, nectar metabolites, antibacterial, homeostasis

## Abstract

Plant specialized metabolites are species-specific compounds that help plants adapt and survive in constantly changing ecological environments. Nectar contains various specialized metabolites, essential for maintaining nectar homeostasis. In this study, we employed high-performance liquid chromatography (HPLC) to compare the sugar composition between spoilage nectar and natural nectar, with further analysis of variations in color, odor, pH, and hydrogen peroxide (H₂O₂) content. Microbial strains in *Camellia reticulata* nectar were isolated and identified using the spread plate method coupled with DNA sequencing. Liquid chromatography-tandem mass spectrometry (LC-MS/MS) was implemented to characterize metabolite differences between spoilage and natural nectars. Subsequent *in vitro* experiments were conducted to validate the effects of screened nectar metabolites on the isolated microbial strains. The results showed that some *C. reticulata* nectar could spoil and deteriorate, which disrupted nectar homeostasis and significantly reduced the pollination efficiency by pollinators. Spoilage nectar had significant differences in color, odor, sugar composition, pH, and H2O2 content compared to natural nectar. The number of microbial species and quantity in spoilage nectar were much higher. The H2O2 content in natural nectar could reach (55.5 ± 1.80) μM, while it was undetectable in spoilage nectar. A total of 15 distinct microbial strains and 364 differential metabolites were isolated and identified from two types of nectar. *In vitro* experiments demonstrated that H2O2 could inhibit all the bacteria in *C. reticulata* nectar except *Serratia liquefaciens*. 12-Methyltetradecanoic Acid inhibited *Bacillus subtilis*, *Curtobacterium flaccumfaciens*, and *Rothia terrae*, and Myristic Acid only inhibited *Rothia terrae*. The nectar metabolites screened in this study had no effect on the nectar specialist yeast *Metschnikowia reukaufii*. In conclusion, the findings of this study revealed that *C. reticulata* nectar regulates the growth of microorganisms through its metabolites to maintain nectar homeostasis and prevent spoilage. This study improves the understanding of the physiological mechanisms of *C. reticulata* in maintaining nectar homeostasis and provides theoretical support for controlling nectar diseases and sustaining the reproductive fitness of *C. reticulata*. Future research could focus on further exploring the complex interactions between different metabolites in *C. reticulata* nectar and a wider range of microorganisms. Moreover, the development of practical applications based on these findings, such as the development of natural preservatives for nectar-related products or the optimization of pollination efficiency in *C. reticulata* cultivation, could be an important area for future exploration.

## 1 Introduction

Almost 87.5% of the over 300,000 flowering angiosperms in the world require animal pollination (Ollerton et al., 2011). Additionally, 87 of the 115 major global food crops (about 76%) depend on animal pollination (Klein et al., 2007), a process supported by the nectar of pollinating plants (Roy et al., 2017). In addition to being rich in sugars, nectar contains various nutrients such as amino acids, proteins, lipids, organic acids, secondary metabolites, vitamins, and minerals (Nicolson, 2022; Roy et al., 2017). These sugars and nutrients, offered by plants as rewards to pollinators, facilitate pollination (Nepi et al., 2018).

Pollinators are not the only organisms that benefit from nectar. This carbohydrate-rich solution also serves as the ideal habitat for microbial growth and reproduction (Álvarez-Pérez et al., 2024). Normally, microorganisms can be introduced into nectar through airborne transmission or by direct contact with pollinators. Once introduced, the microorganisms grow rapidly by utilizing the nutrients available in nectar (Brysch-Herzberg, 2004; de Vega and Herrera, 2013; Pozo et al., 2009). The composition and abundance of nectar microorganisms are influenced by environmental factors (Samuni-Blank et al., 2014) and the species of flower visitors (Morris et al., 2020). Consequently, there are substantial differences in the species and quantities of nectar microorganisms across different plant species.

After colonization, microorganisms can significantly alter nectar’s physical and chemical properties. They have been reported to modify the temperature (Herrera and Pozo, 2010), viscosity (Matiashe et al., 2014), pH, H_2_O_2_ content, sugar composition and concentration (Canto and Herrera, 2012; Good et al., 2014; Lievens et al., 2015; Vannette et al., 2013), amino acid composition and concentration (Kevan et al., 1988; Peay et al., 2012), and odor (Goodrich et al., 2006; Raguso, 2004) of nectar. Some nectar microorganisms have also been reported to increase the nectar’s alcohol content (Nepi, 2017) These physical and chemical changes significantly affect nectar quality and disrupt nectar homeostasis. The changes in nectar characteristics can potentially reduce their attractiveness to pollinators, thereby altering their flower-visiting behavior and decreasing the reproductive fitness of the pollinating plants (Heil, 2011). However, the fact that a vast majority of plant nectar in nature does not deteriorate despite multiple pollinator visits suggests that nectar-secreting plants have evolved a range of biochemical mechanisms to protect nectar from microbial infections, thereby ensuring high-energy rewards for pollinators (Kessler and Baldwin, 2007).

Nectar requires an active defense system to maintain its homeostasis, combat the growth of harmful microorganisms, and protect its nutritional resources from microbial exploitation. Certain nectar traits are believed to exhibit antimicrobial activity. For instance, the high sugar concentrations in nectar create extreme osmotic pressure and high C:N ratios, which limit microbial growth (Herrera et al., 2010; Lievens et al., 2015). Additionally, antibacterial compounds, especially proteins and secondary metabolites, often protect nectar from microbial growth (Schmitt et al., 2021). For example, in response to microbial infection, *Petunia hybrida* nectar can trigger the extensive expression and secretion of S-ribonuclease, peroxidase, and chitinase in the nectaries to counter microbial stress (Hillwig et al., 2011). Similarly, apple nectar chitinase can enhance the resistance of apples to *Erwinia amylovora*, the pathogen responsible for fire blight (Kurilla et al., 2019). With strong antifungal activity, the major nectar protein of *Brassica rapa* is a non-specific lipid transfer protein, BrLTP2.1 (Schmitt et al., 2018).

Some nectar proteins do not directly inhibit bacteria but instead synthesize certain antibacterial substances to combat nectar microorganisms through their own enzyme activity. Tobacco nectar protein can synthesize H_2_O_2_, with concentrations reaching up to 4 mM, thereby protecting tobacco nectar from microbial invasion (Carter and Thornburg, 2004). In 2000, Adler proposed the hypothesis that secondary metabolites in nectar could inhibit the growth of nectar microorganisms (Adler, 2000). Since then, various secondary metabolites have been reported to exhibit antibacterial effects in environments beyond nectar (Barberis et al., 2023; Stevenson et al., 2017). However, direct evidence of the inhibition of nectar microorganisms by secondary metabolites in nectar remains limited (Block et al., 2019; Mueller et al., 2023), and the mechanisms by which nectar maintains microbial balance through secondary metabolites remain largely unclear (Heil, 2011). It is also unknown whether other metabolites of nectar play a role in maintaining nectar homeostasis, such as amino acids, sugars, or lipids.

Plants are natural experts in organic synthesis, being able to generate large numbers of specific metabolites with widely varying structures that help them adapt to variable survival challenges (Shen et al., 2023). As a unique product of plant physiological activities, nectar contains a wide variety of metabolites with complex compositions (Nicolson, 2022; Roy et al., 2017). However, most of the current researches on the antibacterial properties of nectar metabolites focused on a certain category or just a few known substances (Anjali et al., 2023; Fischer, 2020; Mueller et al., 2023). It is difficult to comprehensively understand the whole picture of nectar metabolites, which greatly hinders the further expansion and deepening of relevant research. Metabolomics is a research discipline that integrates the capabilities of several types of research including analytical chemistry, statistics, and biochemistry (Shen et al., 2023). The use of metabolomic methods enables unbiased and comprehensive detection and analysis of various metabolites in nectar, providing strategies for gaining a systematic understanding of quantitative changes in the levels of metabolites. On the one hand, it helps us discover new metabolites that may play a key role in the interaction between nectar and microorganisms. On the other hand, it also opens up a new path for revealing the physiological mechanisms by which nectar metabolites maintain nectar homeostasis.

*C. reticulata* is an evergreen arbor belonging to the genus *Camellia* of the family Theaceae. It is a unique woody oil tree species in Yunnan (Liu et al., 2023). Its seeds have a high oil content and is rich in unsaturated fatty acids, vitamin E, and has the effects of softening blood vessels and reducing blood lipids (Qin et al., 2024). It is also the original species of Yunnan camellia flower, and is of great scientific value for studying the origin, evolution, and genetic diversity of camellia plants (Xin et al., 2017). It is a rare species under second-class national protection of China. *C. reticulata* has the trait of self-incompatibility. The fruit-setting rate through wind-pollination is merely 3.54% (Li et al., 2024). Given that its blooming season occurs in winter, when the number of pollinating insects is extremely low, it mainly depends on birds for pollination. Nectar, a pivotal factor in attracting pollinators to pollinate *C. reticulata*, is central to its reproductive process. However, our investigations have revealed that during the flowering period of *C. reticulata*, issues such as nectar spoilage and premature wilting and dropping of flowers frequently occur. These problems pose a severe threat to the pollination efficiency and fruit-setting rate of *C. reticulata*. Previous research has demonstrated that the excessive proliferation of typical spoilage microorganisms is the primary cause of food spoilage (including nectar spoilage) (Gram et al., 2002; Madan et al., 2024; Vannette et al., 2013). Additionally, some studies have indicated that it is also a significant trigger for the pathological changes in nectary tissues (Wei et al., 1992; Wilson et al., 1990). Therefore, delving deep into the antibacterial mechanism of the nectar of *C. reticulata* holds vital importance for enhancing its pollination efficiency and ultimately boosting its yield.

Previous studies have demonstrated that plant nectar contains abundant antibacterial substances (Schmitt et al., 2021). It is therefore likely that *C. reticulata* must also possess antibacterial substances to maintain the stability of the chemical composition of nectar. However, this area remains unexplored. For this reason, this study mainly focuses on the following aspects: (1) Clarifying the physicochemical changes involved in nectar spoilage; (2) Isolating and identifying the microorganisms in the nectar of *C. reticulata*; (3) Identifying and screening antibacterial substances in the nectar of *C. reticulata*; (4) Examining the inhibitory effect of the antibacterial substances on nectar microorganisms. These findings will not only provide a scientific basis for understanding the physiological mechanisms that maintain nectar homeostasis in *C. reticulata* but also serve as a reference for exploring the interactions among plants, microorganisms, and pollinators.

## 2 Materials and methods

### 2.1 Sampling information and sample collection

*C. reticulata* is a winter flowering plant with a blooming period roughly from mid-November to mid-March next year. The nectar of *C. reticulata* was collected in January 2024 from the germplasm resources conservation bank of *C. reticulata* (98°35’9.366”E, 24°57’25.456”N) at Shaba Forest Farm in Tengchong, Yunnan Province. Clear and odorless natural nectar was collected from healthy and undamaged flowers. Spoilage nectar, identified by its cloudy appearance and sour odor, was collected from flowers exhibiting tissue lesions. The collected nectar samples were transported to the laboratory on dry ice and stored at -80°C for future use.

### 2.2 Determination of the physicochemical properties of natural nectar and spoilage nectar

#### 2.2.1 Measuring nectar color, odor, pH, and hydrogen peroxide content

Through visual observation and olfaction, the color, solution state, and odor of the natural and spoilage nectar were assessed. A pH meter (Sangon Biotech, Shanghai, China) was used to measure the pH of the two types of nectar. H_2_O_2_ concentration was quantified using an H_2_O_2_ content assay kit (Sangon Biotech, Shanghai, China). Briefly, 100 μL of nectar or an H_2_O_2_ standard solution was processed according to the instructions of the reagent kit. The absorbance was measured at 415 nm using a microplate reader (Bio-Rad, CA, USA). The hydrogen peroxide concentration in nectar was calculated using the formula provided in the assay kit’s manual.

#### 2.2.2 Determination of total sugar and sugar components in nectar

Nectar concentration was measured using a Digital sugar refractometer (GAO Tek, Toronto, Canada), with a Brix value in the range of 0% ~ 32%. A sugar standard (National Institute of Metrology, China) was dissolved in ultrapure water to prepare a standard curve solution, which was filtered using a 0.45 μm filter membrane. The nectar sample was diluted with ultrapure water to ensure that the final sugar concentration fell within the standard curve concentration range (0.5 mg/Ml ~ 2.0 mg/mL) and was then filtered with a 0.45μm aqueous filter membrane. The samples were analyzed using a High Performance Liquid Chromatography (HPLC) system (Shimadzu LC-10A). The samples were separated and detected using Shimadzu’s amino column (INERTSIL NH_2_ 5um 4.6 mm × 250 mm) and an evaporative light scattering detector (Evaporation chamber temperature 50°C, gain 4, pressure 350 kPa).

The mobile phase A and B consisted of acetonitrile and water, respectively. The gradient program was as follows: 0 ~ 7 minutes: 20% of B; 7 ~ 8 minutes: Increase B from 20% to 25%; 8 ~ 18 minutes: B maintained at 25%; 18 ~ 19 minutes: Decreased B from 25% to 20%; 19 ~ 27 minutes: Maintained B at 20%. The flow rate, column temperature, and injection volume were set at 1mL/min, 35°C, and 20 μL, respectively. The retention time of the sugars in the samples was compared with the chromatographic peaks of the standards. The sugar content in the sample was calculated based on the standard curve linear regression equation and expressed in mg/mL nectar solution.

### 2.3 Counting, isolation, and identification of nectar microbial culture

1 mL of nectar was diluted in sterile 1 × Phosphate Buffer Saline (PBS) to create serial dilutions ranging from 10 to 10^6^ times. Next, 200 μL of the diluted sample was spread evenly on a Luria-Bertani (LB) agar medium plate, with each dilution repeated in triplicate. The plates were cultured at 30°C for 48 h to 72 h. The plates with colony count between 30 CFU/mL and 300 CFU/mL and no spreading colonies were selected for counting the total number of colonies. The nectar bacterial strains were classified based on their colony morphology. Single colonies were isolated and cultured in LB liquid medium at 30°C for 24 h to 48 h at 220 rpm. The strains were isolated and purified through repeated streaking and subculture based on the colony morphology. Next, the isolated and purified bacterial strains were subjected to 16S rRNA sequencing, while the yeast strains were subjected to the large-subunit ribosomal RNA (LSU rRNA) sequencing. The nectar microbial sequences obtained from 16S rRNA (V1-V9 variable region, Forward primer: 5′-AGAGTTTGATCCTGGCTCAG-3′, Reverse primer: 5′-TACGGCTACCTTGTTACGACT-3′) and LSU rRNA (D1-D2 variable region, Forward primer: 5′-GCATATCAATAAGCGGAGGAAAAG-3′, Reverse primer: 5′-GGTCCGTGTTTCAAGACGG-3′) sequencing were compared with the GenBank database using the BLAST tool on the NCBI website for homology analysis. DNA sequencing was performed on the isolated bacteria using an 3730xl DNA Analyzer (Applied Biosystems, US), following the sequencing method described by Jiang et al. (2006). The microbial species in nectar were identified based on the information of the known species with the highest homology. All obtained sequences have been deposited in GenBank (**Supplementary Table S1**).

### 2.4 Determination of the effect of nectar microorganisms on the pH of the medium

The isolated and purified single colonies were cultured in LB liquid medium at 30°C with shaking at 220 rpm until the Optical Density (OD) value of the culture medium was almost 1.0. The culture liquid was then added into fresh LB liquid medium at a ratio of 1:10 and incubated at 30°C with shaking at 220 rpm for 24 hours. After incubation, the cultures were centrifuged to obtain the supernatant. The pH of the medium was measured before and after adding the bacterial culture.

### 2.5 Widely targeted metabolome identification of nectar metabolites

For both natural nectar and spoilage nectar, three biological replicates are set in each group for widely targeted metabolome analysis.

#### 2.5.1 Nectar metabolites extraction

1mL of nectar was freeze-dried under vacuum followed by the addition of 1000 μL of extraction solution (methanol: acetonitrile: water in a 1:2:1 volume ratio). The samples were vortexed and mixed for 30 seconds. Next, steel balls were added and the mixture was treated with a 45 Hz grinder for 10 minutes, and sonicated in an ice bath for 10 minutes. Following sonication, the sample was allowed to stand at -20°C for one hour, after which it was centrifuged at 4°C and 12000 rpm for 15 minutes. After centrifugation, 300 μL of the supernatant was taken and filtered through a 0.22 μm organic filter membrane into a 2 mL injection bottle. For quality control (QC), 10 μL from each sample was pooled to create QC samples for machine testing.

#### 2.5.2 LC-MS/MS analysis and identification of nectar metabolites

The sample extracts were analyzed using an Ultra Performance Liquid Chromatography-Electrospray Ionization-Tandem Mass Spectrometry (UPLC-ESI-MS/MS) system (UPLC, Waters Acquity I-Class PLUS; MS, Applied Biosystems QTRAP 6500^+^). The following analytical conditions were used: For UPLC, Waters HSS-T3 (1.8 µm, 2.1 mm × 100 mm) column was used. The mobile phase consisted of solvent A-pure water with 0.1% formic acid and 5 mM Ammonium acetate, and solvent B-acetonitrile with 0.1% formic acid. The sample measurements were performed with the following gradient program: Initial 98% A, 2% B for 1.5 min; 1.5min ~ 5.0 min – Linear gradient to 50% A, 50% B.; 5.0 ~ 9.0 min - Linear gradient to 2% A, 98% B and held for 1min; 9.0 ~ 10.0 min - adjusted back to 98% A, 2% B and held for 3min. The flow velocity, column oven temperature, and injection volume were set at 0.35 mL per minute, 50°C, and 4 μL, respectively.

The effluent was alternatively connected to an ESI-triple quadrupole-linear ion trap (QTRAP)-MS. The ESI source operation parameters were as follows: The source temperature was 550°C and the ion spray voltage (IS) was 5500 V (positive ion mode)/-4500 V (negative ion mode). The ion source gases, gas I (GSI), gas II(GSII), and curtain gas (CUR) were set at 50, 55, and 35 psi, respectively. The medium was collision-activated dissociation (CAD). Instrument tuning and mass calibration were performed with 10 and 100 μmol/L polypropylene glycol solutions in Triple Quadrupole (QQQ) and Linear Ion Trap (LIT) modes, respectively. QQQ scans were performed as Multiple Reaction Monitoring (MRM) experiments with nitrogen as the collision gas set to medium. DP (declustering potential) and CE (collision energy) were optimized for individual MRM transitions. A specific set of MRM transitions was monitored for each period according to the metabolites eluted within that period.

#### 2.5.3 Data analysis

The original peak area data was normalized using the total peak area and subsequent analyses were conducted. The Principal component analysis and Spearman correlation analysis were employed to assess the repeatability of the samples within the group and the quantity control samples. The identified compounds were categorized and assigned pathway information using KEGG, HMDB, and LipidMaps databases. The difference multiples were calculated and compared according to the grouping information. A T-test was used to determine the significance of the differences (*p* value) of each compound. The R language package ropls was used to perform Orthogonal Partial Least Squares Discriminant Analysis (OPLS-DA) modeling, and the reliability of these models was evaluated by performing 200 permutation tests. The Variable Importance in Projection (VIP) value of the model was calculated using multiple cross-validation. The method of combining the difference multiple, the *p* value, and the VIP value of the OPLS-DA model was adopted to screen the differential metabolites. The screening criteria were the following: Fold Change (FC) > 1, *p* value < 0.05 and VIP > 1. The differential metabolites of KEGG pathway enrichment significance were calculated using a hypergeometric distribution test.

### 2.6 Validation of the effect of nectar metabolites on nectar microorganisms

Ten nectar metabolites exhibiting significant differences in expression between natural and spoilage nectar and demonstrated inhibitory effects on certain microorganisms (**Table 1**), along with hydrogen peroxide (undetectable in spoilage nectar, **Table 2**) were selected as candidate antibacterial substances. Antibacterial validation experiments were conducted on 15 nectar cultivable microorganisms (**Table 3**). Each strain was isolated, purified, inoculated into an LB liquid medium, and cultured for 24 hours. The cultures were diluted with PBS solution to 0.5 McFarland turbidity (approximately 10^8^ CFU/mL) and diluted 1000-fold to prepare a bacterial suspension of approximately 10^5^ CFU/mL. 100 μL of the diluted bacterial culture was spread evenly on LB agarose plates. After the bacterial suspension dried, an 8 mm sterile puncher was used to punch holes in the agar. Next, 100 μL of 500 μM nectar metabolites or 100 μL of 50 μM hydrogen peroxide were added to the holes to examine antibacterial activity. Refer to the minimum inhibitory concentration (MIC) determination method established by Andrews (2001) to determine the MIC of nectar metabolites.

**Table 1:** Nectar metabolite used for antibacterial validation.

Metabolite	Category	Molecular formula	Molecular Weight (g/mol)	Microorganism strains	Ref.
Artemisic Acid	Terpenoids	C_15_H_22_O_2_	234.33	*Escherichia coli* *Pseudomonas aeruginosa* *Staphylococcus aureus* *Mycobacterium smegmatis* *Mycobacterium smegmatis* mc^2^ 155 *Staphylococcus aureus* (MRSA)	(Bhowmick et al., 2020)
(+)-Catechin Hydrate	Flavonoids	C_15_H_16_O_7_	308.28	*Escherichia coli* *Pseudomonas aeruginosa* *Streptococcus mutans*	(Wu and Brown, 2021)
Jasmonic acid	Lipid	C_12_H_18_O_3_	210.27	*Pyricularia oryzae*	(Neto et al., 1991)
D-(+)-Phenyllactic Acid	Organic acid	C_9_H_10_O_3_	166.17	*Staphylococcus aureus* *Staphylococcus epidermidis* *Listeria monocytogenes* UCMAL205 *Providencia stuartii* *Klebsiella oxytoca* *Aeromonas hydrophila*	(Dieuleveux et al., 1998)
Ferulic Acid	Organic acid	C_10_H_10_O_4_	194.18	*Escherichia coli* *Pseudomonas aeruginosa* *Staphylococcus aureus*, *Listeria monocytogenes*	(Borges et al., 2013)
Myristic Acid	Lipid	C_14_H_28_O_2_	228.37	*Listeria monocytogenes* *Staphylococcus epidermidis*	(Chen et al., 2019) (Liu and Huang, 2012)
Quercetin 3-D-galactoside	Flavonoids	C_21_H_20_O_12_	464.4	*Colletotrichum gloeosporioides*	(Sudheeran et al., 2020)
Ruscogenin	Terpenoids	C_27_H_42_O_4_	430.6	*Staphylococcus aureus* *Bacillus cereus* *Escherichia coli* *Pseudomonas aeruginosa* *Candida albicans* *Aspergillus niger*	(Nazemiyeh et al., 2020)
12-Methyltetradecanoic Acid	Lipid	C_15_H_30_O_2_	242.40	*Pseudomonas aeruginosa* PAO1 *Magnaporthe oryzae*	(Inoue et al., 2012) (Jeon et al., 2010)
Crebanine	Alkaloids	C_20_H_21_NO_4_	339.4	*Escherichia coli* *Pseudomonas aeruginosa* *Staphyloccocus aureus* *Proteus vulgaris* *Salmonella typhi* *Shigella dysenteriae* *Micrococcus lysodeikticus* *Bacillus cereus* *Bacillus megaterium* *Bacillus subtilis* *Cercospora kaki* *Gymnosporangium haraeanum* *Pyricularia oryzae* *Rhizoctonia solani* *Colletotrichum graminicola*	(Deng et al., 2011)

**Table 2:** Comparison of the physical and chemical properties between natural nectar and spoilage nectar.

Index	Natural nectar	Spoilage nectar
Color	colorless and clear	brown and turbid
Odor	faint scent	pungent and sour smell
pH	5.13 ± 0.06^a^	3.23 ± 0.08^b^
H_2_O_2_ content(uM)	55.5 ± 1.80^a^	0.00 ± 0.00^b^
Total sugar Brix(%)	26.10 ± 2.14^a^	28.20 ± 1.31^b^
Sucrose(mg/mL)	100.04 ± 3.53^a^	108.93 ± 4.42^b^
Glucose(mg/mL)	9.54 ± 0.33^a^	10.66 ± 1.58^b^
Fructose(mg/mL)	9.01 ± 0.16^a^	8.66 ± 0.12^b^
Maltose(mg/mL)	19.40 ± 0.04^a^	14.1 ± 0.05^b^

All data are mean ± SD, SD, Standard Deviation; Different letters on the same row indicate significant differences, *p*<0.05.

**Table 3:** Validation of the inhibitory effect of nectar substances on cultivable microorganisms in nectar (mean ± SD).

Strains	Microorganisms	H_2_O_2_		12-Methyltetradecanoic Acid		Myristic Acid	
		Inhibition zone diameter (mm)^a^	MIC (uM)	Inhibition zone diameter (mm)^a^	MIC (uM)	Inhibition zone diameter (mm)^a^	MIC (uM)
	Gram-negative bacteria						
Na-5	*Agrobacterium rosae*	29.17 ± 0.45	6.4	NI	/	NI	/
S-1	*Xanthomonas hydrangeae*	34.00 ± 0.88	3.2	NI	/	NI	/
S-2	*Pantoea agglomerans*	37.10 ± 0.50	3.2	NI	/	NI	/
S-4	*Lelliottia amnigena*	28.08 ± 0.51	6.4	NI	/	NI	/
S-7	*Erwinia amylovora*	28.07 ± 0.25	6.4	NI	/	NI	/
S-8	*Rosenbergiella epipactidis*	30.13 ± 1.10	6.4	NI	/	NI	/
S-10	*Xanthomonas campestris pv. raphani*	35.23 ± 1.46	3.2	NI	/	NI	/
S-11	*Serratia liquefaciens*	NI	/	NI	/	NI	/
S-12	*Pseudomonas palleroniana*	26.97 ± 0.70	12.8	NI	/	NI	/
S-13	*Xanthomonas arboricola pv. pruni*	35.3 ± 1.25	3.2	NI	/	NI	/
	Gram-positive bacteria						
Na-3	*Bacillus subtilis*	26.27 ± 0.80	12.8	10.33 ± 0.32	256	NI	/
S-3	*Rothia terrae*	50.07 ± 1.25	1.6	11.17 ± 0.25	256	14.23 ± 0.72	64
S-5	*Curtobacterium flaccumfaciens*	27.93 ± 1.19	12.8	12.20 ± 0.36	128	NI	/
S-9	*Leuconostoc suionicum*	30.07 ± 1.85	6.4	NI	/	NI	/
	Yeast						
Y1	*Metschnikowia reukaufii*	NI	/	NI	/	NI	/

NI, no Inhibition zone; / indicates no data; ^a^Well diameter, 8 mm.

### 2.7 Statistic analysis

The mean and standard deviation of the pH of nectar and LB medium, sugar concentration, and nectar microbial population were calculated using SPSS 19 (SPSS Inc., Chicago, IL, USA). A *p* value < 0.05 was considered to be a statistically significant difference.

## 3 Results

### 3.1 Differences in the physicochemical properties of natural nectar and spoilage nectar

**Figure 1:**
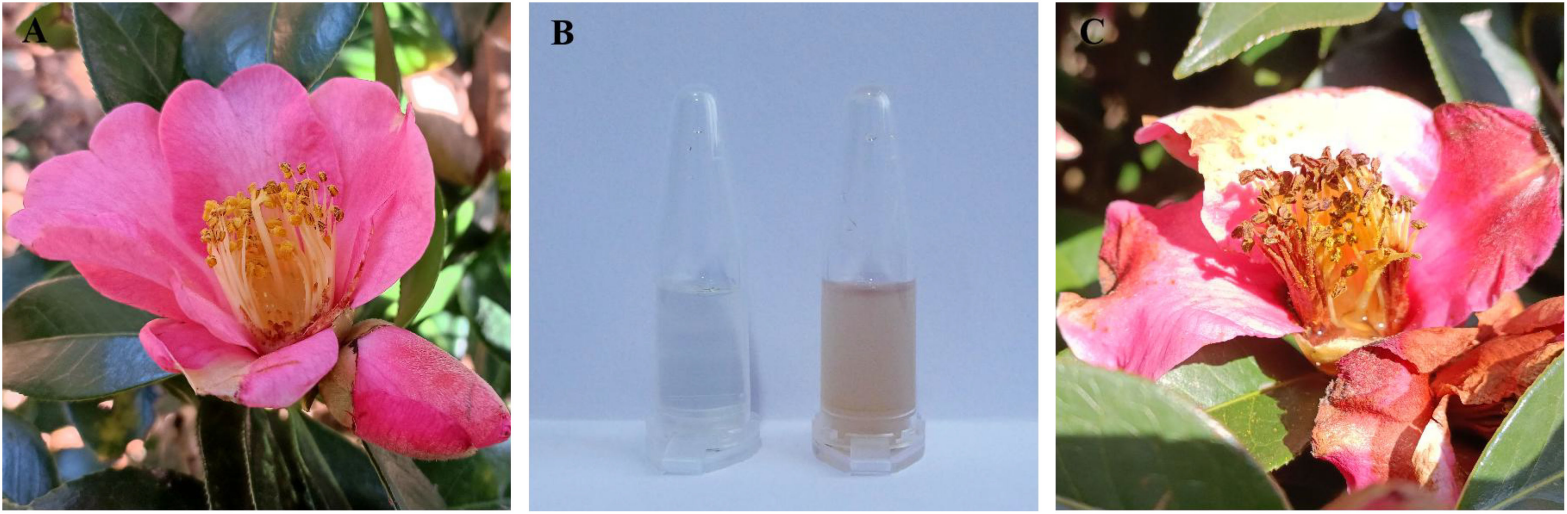
Comparison of natural nectar and spoilage nectar in *C reticulata*. **(A)** Natural nectar within the flowers; **(B)** Collected natural nectar (left) and spoilage nectar (right); **(C)** Spoilage nectar within the flowers.

As shown in **Figure 1**, the natural nectar of *C. reticulata* is a colorless, transparent, and clear liquid, whereas spoilage nectar appears as a brown, opaque, and turbid liquid. Nectar spoilage results in a sour and pungent odor, which is accompanied by a decrease in H_2_O_2_ and pH. Additionally, it increases the total sugar, sucrose, and glucose contents, whereas decrease fructose and maltose levels (**Table 2**).

### 3.2 Influence of nectar spoilage on flower tissue

**Figure 2:**
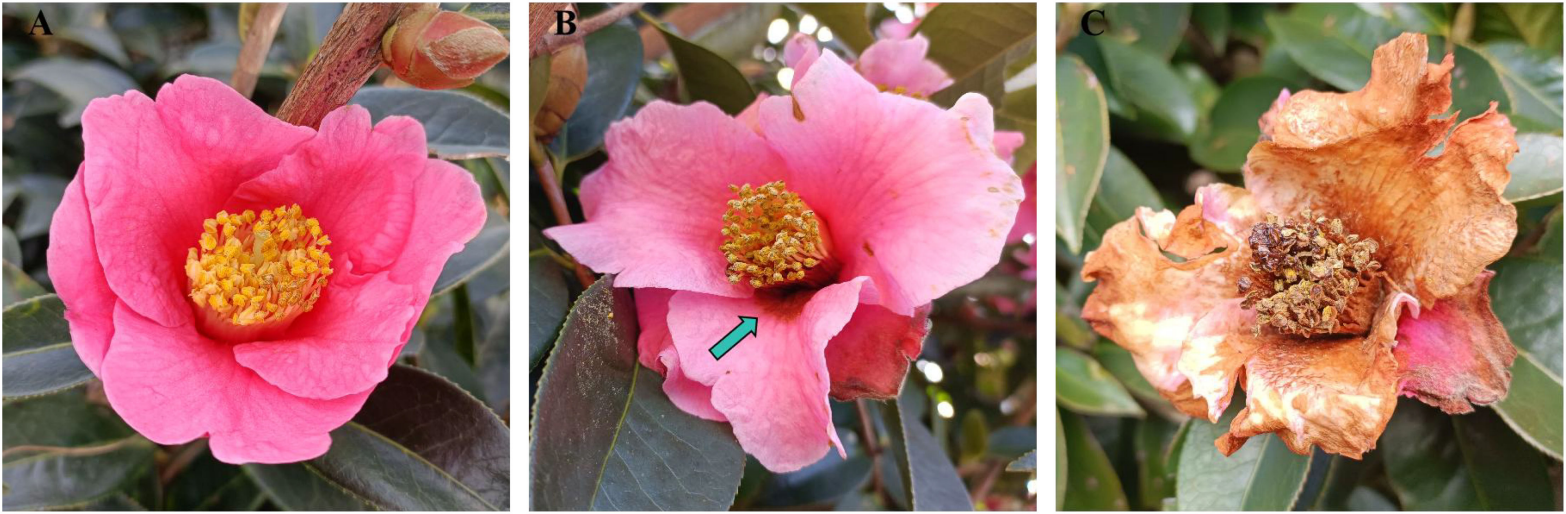
Effects of nectar spoilage on the flowers of *C reticulata*. **(A)** Healthy flower showing no lesions; **(B)** Flower showing early-stage lesions; **(C)** End stage of flower disease.

The flowers of *C. reticulata* are bell-shaped, with pink petals, numerous stamens, and yellow anthers (**Figure 2A**). However, when the microorganisms proliferate excessively in the nectar of *C. reticulata*, the nectar rots and deteriorates. As shown in **Figure 2B**, pathogenic microorganisms would invade the flower tissue via the nectarthodes at the bottom of the filament, leading to flower tissue decay that begins at the base of the filament and gradually spreads throughout the flower. This process shortens the flower’s lifespan, causing it to wither prematurely (**Figure 2C**). Furthermore, spoilage nectar emits an unpleasant odor, deterring pollinators, such as bees and birds, thereby hindering the spread of pollen.

### 3.3 Counting the microbial number of natural and spoilage nectar in *C. reticulata*

The number of microorganisms in natural nectar was determined to be (2.28 ± 0.97) × 10^4^ CFU/mL, using the plate colony counting method, while the number of microorganisms in spoilage nectar was (1.72 ± 0.61) × 10^8^ CFU/mL. Evidently, the number of microorganisms in spoilage nectar was significantly higher than in natural nectar, *p* < 0.05.

### 3.4 Identifying the cultivable microorganisms in natural nectar and spoilage nectar

Six bacterial strains (Na-1 ~ Na-6) and one yeast strain (Y-1) were isolated from natural nectar, while 13 bacterial strains (S-1 ~ S-13) and one yeast strain (Y-2) were isolated from spoilage nectar. The 16S rRNA sequences of bacterial strains and the LSU rRNA sequences of yeast strains were analyzed using the BLAST tool on the NCBI website. The identified sequences were compared with the GeneBank database for homology analysis and the microbial species of nectar were determined based on the information of the known species with the highest homology (**Table 4**). Six bacterial strains from natural nectar were identified, representing 4 genera and 4 species, while 13 bacterial strains from spoilage nectar were identified, belonging to 10 genera and 12 species. *Pseudomonas palleroniana* and *Rothia terrae* were identified in both natural and spoilage nectar. The yeast species in both types of nectar was the nectar specialist yeast *M. reukaufii*. According to the BacDive database (https://bacdive.dsmz.de/), 7 bacterial species isolated from spoilage nectar were identified as plant pathogens, including *Curtobacterium flaccumfaciens*, *Erwinia. amylovora*, *Lelliottia. amnigena*, *Pseudomonas palleroniana*, *Xanthomonas. arboricola pv. Pruni*, *Xanthomonas. campestris pv. Raphani* and *Xanthomonas. hydrangeae*.

**Table 4:** Identification of culturable microorganisms present in the nectar of *C. reticulata*.

Strains	Species	Sequencing Length (bp)	Accession number	Closest number	Identity (%)
Na-1	*Pseudomonas palleroniana*	1364	PQ722026	PQ113825.1	100%
Na-2	*Rothia terrae*	1248	PQ722027	MW577404.1	100%
Na-3	*Bacillus subtilis*	1391	PQ722028	KU358720.1	99.93%
Na-4	*Rothia terrae*	1375	PQ722029	MT180559.1	99.93%
Na-5	*Agrobacterium rosae*	1328	PQ722030	NR_178307.1	99.85%
Na-6	*Rothia terrae*	1375	PQ722031	EF540463.1	100%
S-1	*Xanthomonas hydrangeae*	1387	PQ722032	NR_181958.1	100%
S-2	*Pantoea agglomerans*	1364	PQ722033	MH997440.1	99.78%
S-3	*Rothia terrae*	1338	PQ722034	MW577404.1	100%
S-4	*Lelliottia amnigena*	1399	PQ722035	KT767882.1	99.93%
S-5	*Curtobacterium flaccumfaciens*	1370	PQ722036	MN989053.1	99.93%
S-6	*Xanthomonas arboricola pv. pruni*	1406	PQ722037	MF351917.1	99.93%
S-7	*Erwinia amylovora*	1399	PQ722038	CP076594.1	99.14%
S-8	*Rosenbergiella epipactidis*	1374	PQ722039	MT341870.1	99.49%
S-9	*Leuconostoc suionicum*	1381	PQ722040	LC782031.1	99.93%
S-10	*Xanthomonas campestris pv. raphani*	1406	PQ722041	CP066930.1	100%
S-11	*Serratia liquefaciens*	1379	PQ722042	MT995042.1	100%
S12	*Pseudomonas palleroniana*	1374	PQ722043	PP758412.1	100%
S13	*Xanthomonas arboricola pv. pruni*	1404	PQ722044	MF351917.1	100%
Y-1	*Metschnikowia reukaufii*	521	PQ721930	EU439452.1	100%
Y-2	*Metschnikowia reukaufii*	524	PQ721931	EU439452.1	99.81%

Markings in the same color indicate that the identification results are of the same species within the same group of nectar.

### 3.5 Influence of nectar microorganisms on the pH of the medium

While natural nectar is inherently acidic, spoilage nectar has even lower acidity (**Table 2**), likely due to the presence of the nectar-inhabiting microorganisms. Measuring the pH of the LB media before and after adding microbial cultures revealed that most nectar isolates can lower the pH of the medium. Particularly, *Leuconostoc suionicum* and *M. reukaufii* reduced the pH of the culture medium by almost 3 pH units (**Table 5**).

**Table 5:** Changes in pH of culture medium before and after microbial cultivation (mean ± SD).

Strains	Species	pH of the culture medium before cultivation	pH of the culture medium after cultivation	*p* value
Na-1	*Pseudomonas palleroniana*	7.01 ± 0.01	7.05 ± 0.04	>0.05
Na-2	*Rothia terrae*	7.01 ± 0.01	5.20 ± 0.08	<0.05
Na-3	*Bacillus subtilis*	7.01 ± 0.01	5.51 ± 0.10	<0.05
Na-4	*Rothia terrae*	7.01 ± 0.01	5.20 ± 0.07	<0.05
Na-5	*Agrobacterium rosae*	7.01 ± 0.01	6.13 ± 0.08	<0.05
Na-6	*Rothia* sp.	7.01 ± 0.01	5.16 ± 0.05	<0.05
S-1	*Xanthomonas hydrangeae*	7.01 ± 0.01	7.03 ± 0.07	>0.05
S-2	*Pantoea agglomerans*	7.01 ± 0.01	6.17 ± 0.08	<0.05
S-3	*Rothia terrae*	7.01 ± 0.01	5.19 ± 0.06	<0.05
S-4	*Lelliottia amnigena*	7.01 ± 0.01	6.51 ± 0.13	<0.05
S-5	*Curtobacterium flaccumfaciens*	7.01 ± 0.01	6.20 ± 0.11	<0.05
S-6	*Xanthomonas arboricola pv. pruni*	7.01 ± 0.01	7.02 ± 0.11	>0.05
S-7	*Erwinia amylovora*	7.01 ± 0.01	6.00 ± 0.10	<0.05
S-8	*Rosenbergiella epipactidis*	7.01 ± 0.01	6.21 ± 0.09	<0.05
S-9	*Leuconostoc suionicum*	7.01 ± 0.01	4.19 ± 0.13	<0.05
S-10	*Xanthomonas campestris pv. raphani*	7.01 ± 0.01	7.09 ± 0.06	>0.05
S-11	*Serratia liquefaciens*	7.01 ± 0.01	6.43 ± 0.09	<0.05
S12	*Pseudomonas palleroniana*	7.01 ± 0.01	7.04 ± 0.04	>0.05
S13	*Xanthomonas arboricola pv. pruni*	7.01 ± 0.01	7.05 ± 0.04	>0.05
Y-1	*Metschnikowia reukaufii*	7.01 ± 0.01	4.02 ± 0.08	<0.05
Y-2	*Metschnikowia reukaufii*	7.01 ± 0.01	3.75 ± 0.13	<0.05

### 3.6 Comparative analysis of differential metabolites between spoilage nectar and natural nectar

**Figure 3:**
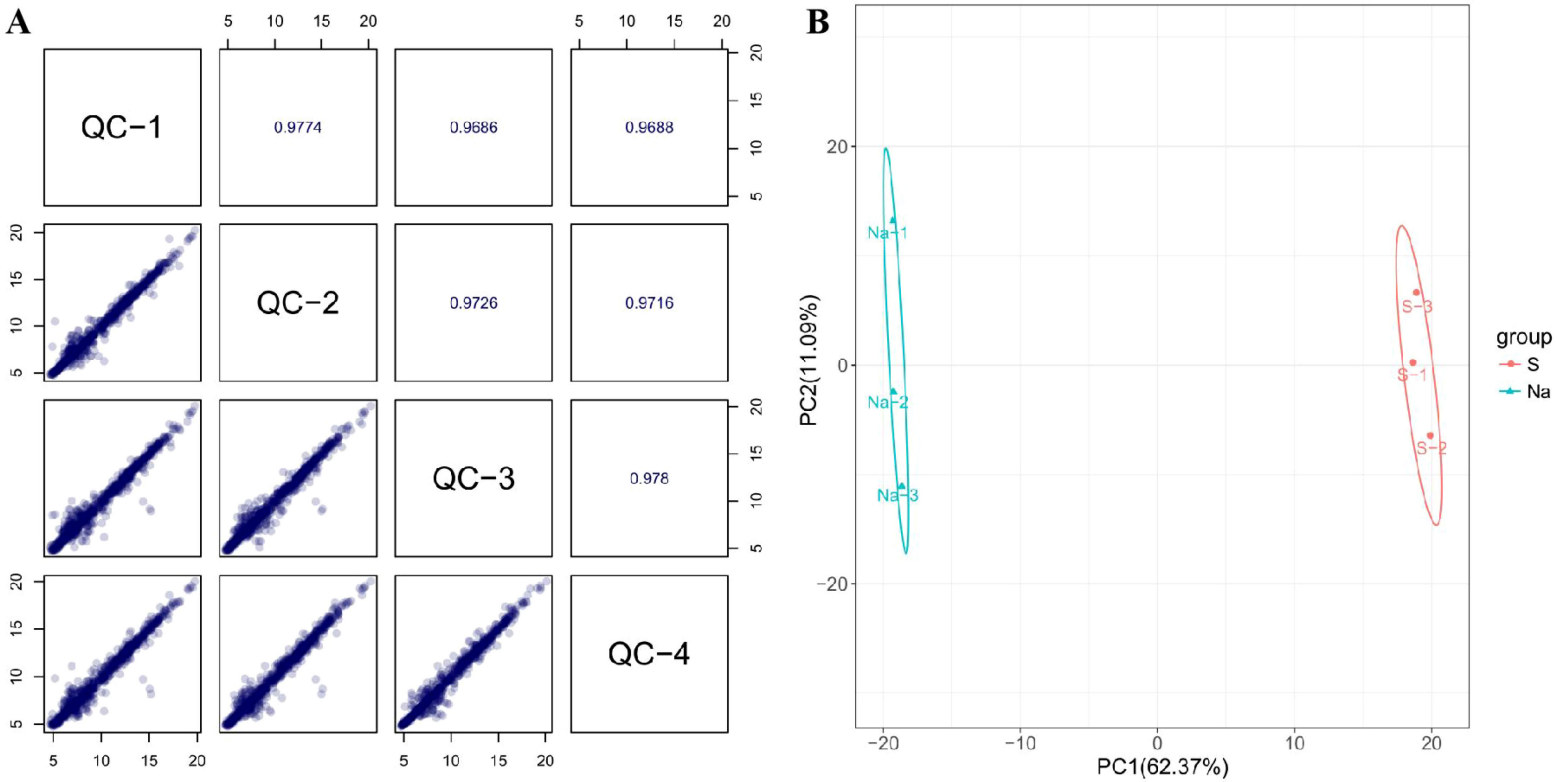
Quality-control analysis of nectar metabolome **(A)** and PCA of *C reticulata* nectar samples from different experimental groups **(B)**.

All of the Spearman’s rank correlation coefficient among different QC samples are above 0.96, indicating a high correlation among these QC samples. Additionally, most of the points (the peak intensity of a QC metabolite) in the scatter plots are distributed along the diagonal, also indicating a good correlation among these QC samples (**Figure 3A**). These imply that the experimental data is of reliable quality, the stability and reproducibility of the instrument analysis are good, and the obtained data is highly credible and can be used for subsequent data analysis. PCA was initially conducted on the entire metabolome data set, to explore clustering in the samples. PCA score plots for nectar samples (**Figure 3B**) showed significant clustering of the natural nectar group (Na) and spoilage nectar group (S), indicating that samples from the spoilage group deviated from normal levels and exhibited metabolic disorders.

**Figure 4:**
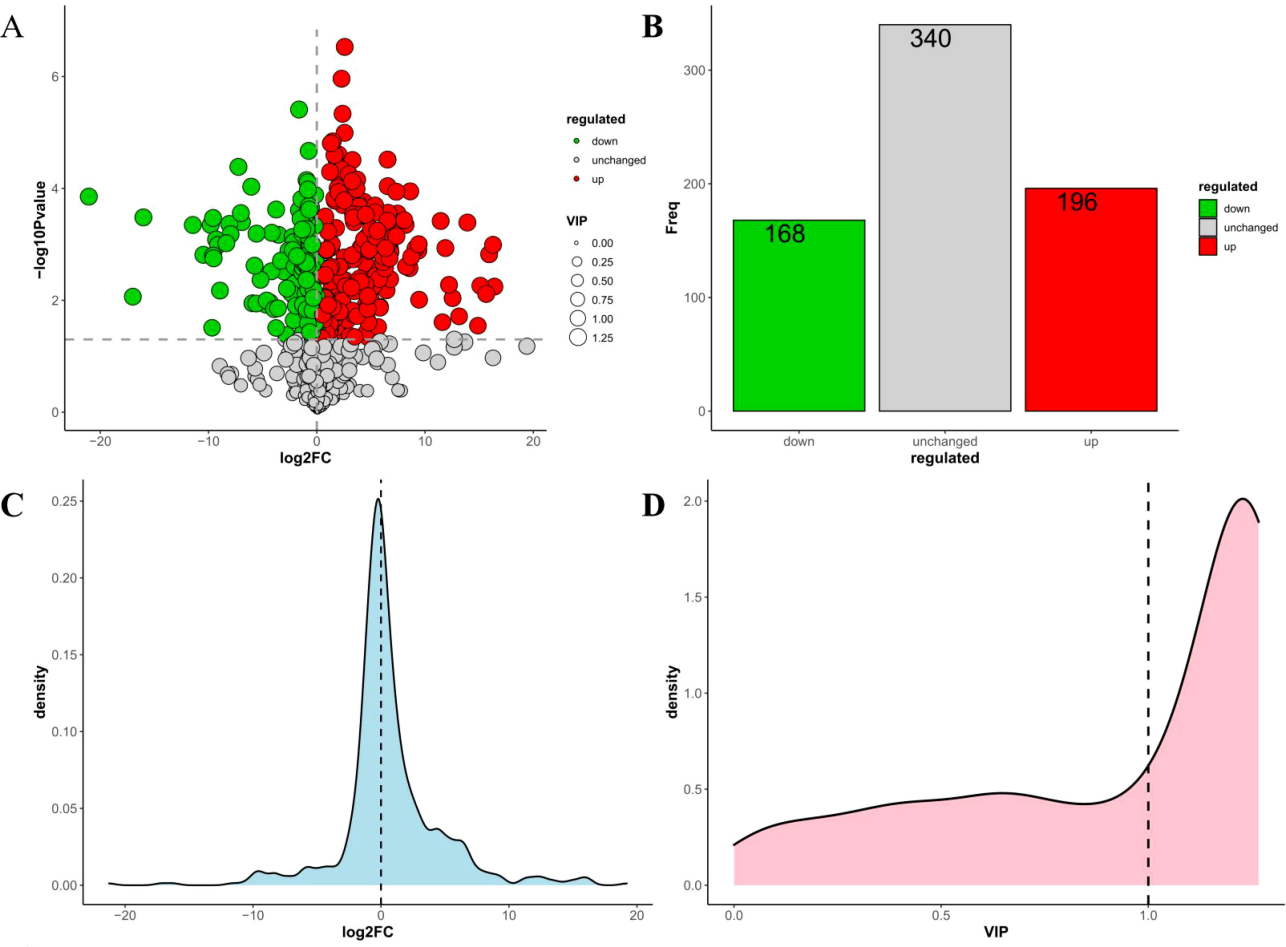
Statistical analysis of differential metabolites in the *C reticulata* nectar. **(A)** Volcanic map of differential metabolites; **(B)** Columnar statistical chart of differential metabolites; **(C)** Log2FC density distribution map of differential metabolites; **(D)** VIP distribution map of differential metabolites.

**Figure 5:**
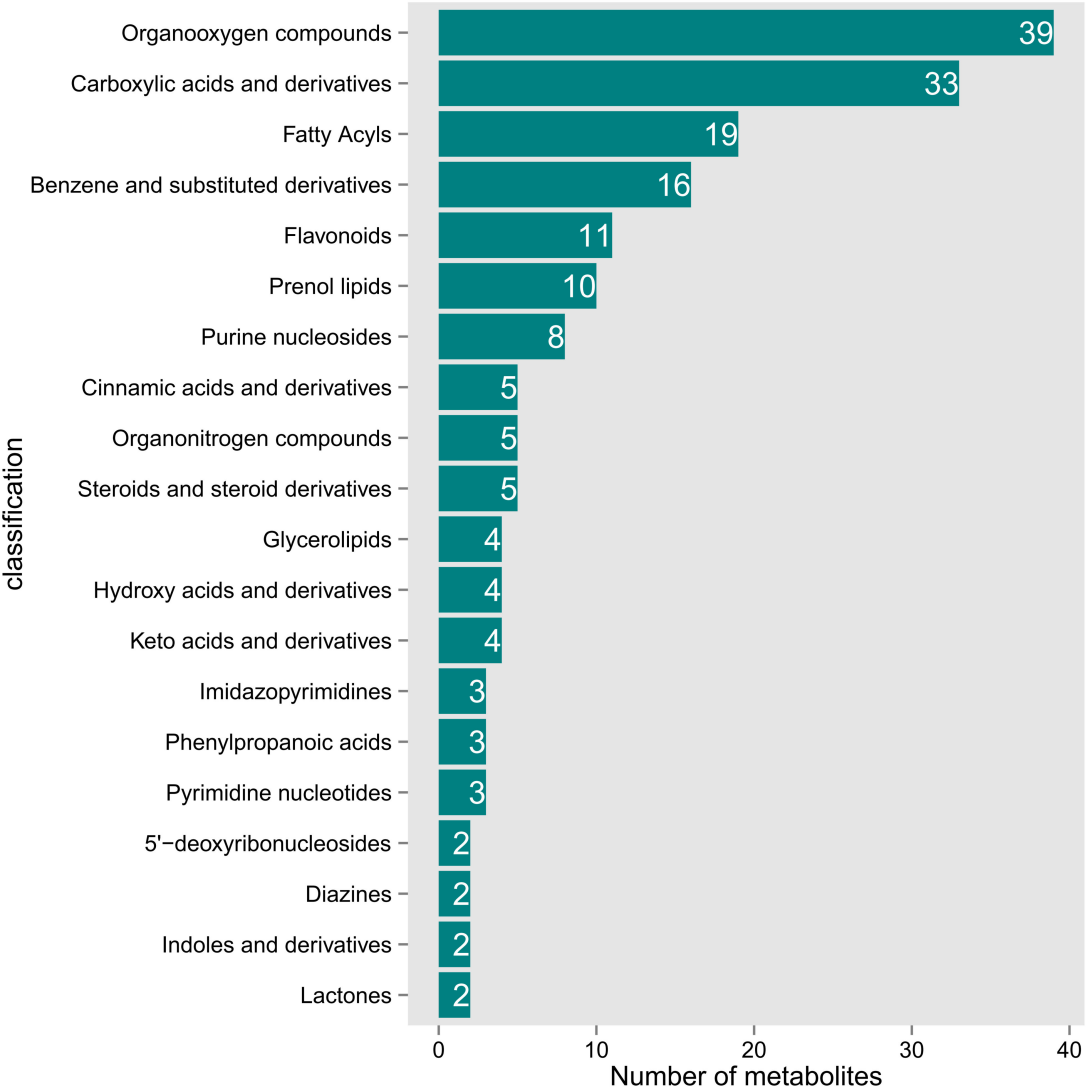
Classification map of the top 20 major differential metabolites in *C. reticulata* nectar.

**Figure 6:**
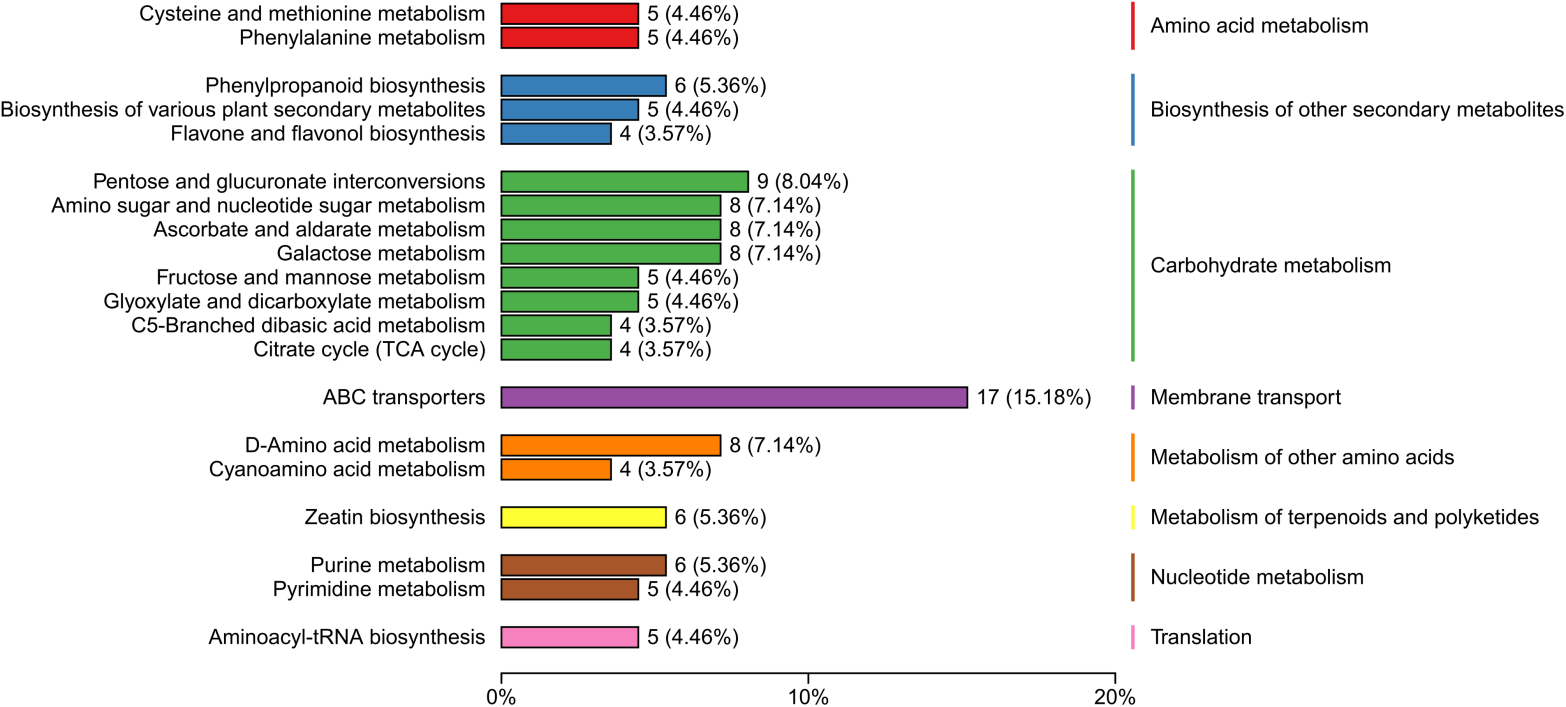
Major pathways associated with the differential metabolites of *C. reticulata* nectar, as revealed by KEGG database annotations.

This study detected 704 metabolites in nectar, with 364 metabolites showing differential expression between spoilage nectar and natural nectar (See **Supplementary Table S2** for details of 364 differential metabolites). Of these, 196 metabolites exhibited increased levels in spoilage nectar, while 168 metabolites showed reduced levels compared to natural nectar (**Figure 4**). As shown in **Figure 5**, the differential metabolites were primarily categorized into organooxygen compounds, carboxylic acids and derivatives, fatty acyls, benzene and substituted derivatives, flavonoids, prenol lipids, and others. Furthermore, KEGG analysis categorized the differential metabolites into three primary metabolic pathways: Environmental Information Processing, Genetic Information Processing, and Metabolism. Additionally, 13 secondary metabolic pathways were identified, including but not limited to amino acid metabolism, biosynthesis of other secondary metabolites, and carbohydrate metabolism. Moreover, 74 tertiary metabolic pathways were found, including cysteine and methionine metabolism, Biosynthetic of various plant secondary metabolites, Pentose and gluconate interconversion and among others (**Figure 6**). The differential metabolites include a range of secondary metabolites, lipids, and antibiotics, which likely play a key role in nectar’s resistance to environmental stress and pathogenic microbial invasion.

### 3.7 Verifying the effect of nectar metabolites on nectar microorganisms

The antibacterial zone diameter and MIC values were used to evaluate the inhibitory effects of 11 nectar metabolites, including H_2_O_2_, on 15 microbial strains isolated from the nectar of *C. reticulata*. The results are illustrated in **Table 3**. H_2_O_2_ inhibited all cultivable nectar bacteria except *S. liquefaciens*. With a MIC of 1.6μM, it exhibited the strongest inhibitory effect on *R. terrae*. Among the tested metabolites, 12-Methyltetradecanoic Acid showed an inhibitory effect on *B. subtilis*, *C. flaccumfaciens*, and *R. terrae*, while Myristic Acid only inhibited *R. terrae*. However, none of the three substances had an inhibitory effect on *M. reukaufii*. Additionally, no inhibitory effects on cultivable nectar microorganisms were detected for the other 8 nectar metabolites.

## 4 Discussion

Nectar homeostasis is crucial for plant pollination (Nepi and Stpiczyńska, 2008). On the other hand, nectar spoilage inevitably loses the plant’s attractiveness to pollinators, ultimately leading to pollination failure. Consequently, understanding nectar ecology is fundamental to advancing pollination biology. Typically, nectar microorganisms are rarely beneficial to plants (Vannette, 2020). Microorganisms entering nectar are controlled by numerous biotic and abiotic factors, making it a multi-dimensional interaction web. Disruptions to any of these factors can destabilize these long-evolved plant-pollinator relations and the intricate interaction network involving multiple communities (Madan et al., 2024). Nectar spoilage is more likely caused by nectar bacteria than by nectar yeast (Vannette et al., 2013). Four bacterial species were isolated from the natural nectar of *C. reticulata*, while twelve species were identified in its spoilage nectar. Furthermore, the number of cultivable bacteria in spoilage nectar was (1.72 ± 0.61) × 10^8^ CFU/mL, significantly higher than that in natural nectar, which was (2.28 ± 0.97) × 10^4^ CFU/mL. Thus, both the diversity and the abundance of bacteria in spoilage nectar greatly exceeded that in natural nectar, which may be the main reason for the spoilage of *C. reticulata* nectar. Additionally, The excessive proliferation of pathogenic bacteria is likely the primary incentive for the nectary tissue lesion of *C. reticulata*. Pathogens can exploit the nectarthodes in nectaries to enter the plant body and cause plant diseases. For example, *E. amylovora*, the causative agent for fire blight, can grow in the nectar of various plant species, such as apples and pears. From there it can enter the nectaries to infect the plant vascular system, causing fire blight (Wei et al., 1992; Wilson et al., 1990). Furthermore, various plant pathogens have been identified in spoilage nectar, including *C. flaccumfaciens*, which causes wilting in dry beans (Osdaghi et al., 2020), and *L. amnigena* and *P. palleroniana*, both of which are known to cause potato soft rot (Osei et al., 2022). Similarly, *X. arboricola pv.Pruni* causes bacterial spot disease on peach trees (Garita-Cambronero et al., 2018) and is listed as a quarantine agent by the European Union, while *X. campestris pv. Raphani* and *X. hydrangeae* are reported to cause leaf spot disease in plants (Campigli and Rizzo, 2023; Cruz et al., 2015). These pathogens may contribute to the premature rotting and withering of *C. reticulata* flowers. However, this hypothesis is speculative based on the pathogenicity of the cultivable bacteria isolated from *C. reticulata* nectar. Further research is necessary to determine whether these pathogens are directly responsible for nectar spoilage and flower tissue lesions.

Nectar serves as the primary source of carbohydrates for pollinators and defensive symbionts. In varying proportions, sucrose, glucose, and fructose are the main solutes found in nectar (Chalcoff et al., 2006). Within a given species, the sucrose-to-hexose ratio and the total sugar concentration are relatively consistent and play a significant role in plant interactions (Petanidou, 2005). Flower visitors have been reported to prefer nectar with a certain sugar ratio. For example, hummingbirds, butterflies, moths, and long-tongued bees typically prefer nectar rich in sucrose, while short-tongued bees and flies favor nectar rich in hexose (Heil, 2011). Additionally, visitors also choose plants based on factors, such as color, aroma, and the taste of nectar (Vannette et al., 2013; Yan et al., 2016; Zhang et al., 2012). The presence of microorganisms in nectar has been demonstrated to reduce sugar concentration, change sugar ratios and contribute to nectar spoilage (Canto and Herrera, 2012; Thompson, 2009). Interestingly, the concentration of total sugar, sucrose, and glucose in the spoilage nectar of *C. reticulata* is higher than that of natural nectar. This difference is likely due to the different sugar preferences of the microorganisms involved in nectar spoilage or the production of extracellular polysaccharides in certain microbial metabolic processes (Bazaka et al., 2011). Another possible explanation for the difference in sugar content between spoilage and natural nectar is that nectar secretion is a dynamic process (Zhang et al., 2023). As nectar spoilage leads to nectary tissue lesions, fresh nectar cannot be replenished in time. Consequently, the nectar becomes more concentrated due to water evaporation from the plant, which occurs when nectar is exposed to environmental factors like heat or air. In addition, the metabolic activity of the microbes that inhabit nectar also affects other nectar traits, such as pH, color, and odor (Rering et al., 2018; Vannette et al., 2013). These changes all can potentially reduce the pollinators’ access to plants (Heil, 2011). As shown in **Table 5**, most microorganisms in the nectar of *C. reticulata* can reduce the pH of the medium. Particularly, *L. suionicum* and *M. reukaufii* significantly reduce the pH of the medium, which is likely the key factor contributing to the lowering of the pH of spoilage nectar. *C. reticulata*, is a cross-pollinated plant that primarily relies on birds for pollination. Upon spoilage, the sugar composition, color, and odor of the nectar change significantly (**Table 2**). These alterations reduce the nectar’s attractiveness to pollinators, ultimately decreasing the pollination efficiency in *C. reticulata* plants.

Not all microorganisms that reach flowers are able to survive and colonize nectar, as they must overcome several layers of barriers. To begin with, there are physical barriers. The sugars (such as sucrose, glucose, and fructose), amino acids, and other small molecules present in nectar regulate its osmotic pressure. A high osmotic pressure environment can forcibly filter out microorganisms that enter nectar (Herrera et al., 2010). Changes in various small-molecule sugars and amino acids in spoilage nectar will surely disrupt the osmotic balance of the nectar (**Supplementary Table S2**). Second, there are biological barriers. Nectar is not a sterile environment and typically contains bacteria and fungi even before the flower buds open (Aleklett et al., 2014). Each species of nectar has its own specific microbial population (Fridman et al., 2012), with competitive exclusion favoring early arriving, faster growing, or inhibiting species (Fukami, 2015). Some microorganisms may be beneficial to nectar. For example, the metabolites of *B. subtilis* can inhibit the growth of *M. reukaufii* (Meng et al., 2024). We isolated *B. subtilis* from natural nectar, but not from spoilage nectar. The absence of Bacillus subtilis in spoilage nectar may have caused the nectar to lose a factor that inhibits the growth of *M. reukaufii*, accelerating the spoilage of the nectar. Lastly, there is a chemical barrier. In addition to being rich in various sugars, nectar contains several other substances, such as secondary metabolites, lipids, and reactive oxygen species, many of which have demonstrated antibacterial properties (Anjali et al., 2023; Fischer, 2020; Mueller et al., 2023). However, direct evidence of the antibacterial effects of these substances in nectar is still limited, and research on *C. reticulata* in this area is scarce.

In the process of nectar maintaining homeostasis, not only the proportions and contents of major components such as sugars need to remain relatively stable, but also the contents and types of secondary metabolites such as flavonoids, polyphenols, terpenoids and alkaloids in the nectar need to be kept in a stable state. These secondary metabolites have functions such as antioxidant (Lang et al., 2024; Shen et al., 2022) and antibacterial (Barati and Modarresi Chahardehi, 2023; Sharma et al., 2020) properties, playing an important role in protecting nectar from microbial invasion and oxidative damage. In *C. reticulata*, compared with the nectar in its natural state, the number of microorganisms in the spoilage nectar increases exponentially. According to the data in **Supplementary Table S2**, most of the flavonoids and polyphenols in the spoiled nectar show an upward trend, which may be related to the removal of superoxide radicals generated by the excessive reproduction of microorganisms in the nectar (He et al., 2023). However, most of the secondary metabolites of terpenoids and alkaloids with antibacterial activity, such as Ruscogenin and Crebanine in **Table 1**, showed a significant downward trend. The decrease in the content of these secondary metabolites is likely to be a key factor accelerating the spoilage of nectar.

The findings of this study revealed 364 differential metabolites between spoilage nectar and natural nectar (**Figure 3**). Among these, there were 120 secondary metabolites and 39 lipids. Quantitative analysis revealed that the H_2_O_2_ content in natural nectar was (55.5 ± 1.80) μM, but it was undetectable in spoilage nectar. As shown in **Table 1**, ten metabolites with known antibacterial activity, along with H_2_O_2_, were selected to investigate their effects on the microorganisms present in the nectar of *C. reticulata*. The results (see **Table 3**) indicated that H_2_O_2_ is the main antibacterial substance in the nectar of *C. reticulata*, capable of inhibiting most nectar microorganisms, though not all. This result is consistent with the findings of Mueller et al. ‘s study on antibacterial compounds in nectar (Mueller et al., 2023). Meanwhile, metabolites such as 12-Methyltetradecanoic Acid, Myristic Acid, and other nectar antibacterial substances (some identified but not yet verified) work alongside H_2_O_2_ to help maintain the nectar homeostasis of *C. reticulata*. The antibacterial effect of nectar metabolites represents an important defense mechanism formed by plants during long-term evolution. Through the synergistic effect of various antibacterial metabolites, this system plays a key role in ensuring plant reproduction and maintaining ecological balance. Future research should focus on exploring the synthesis and regulation mechanisms of antibacterial metabolites in nectar, the co-evolutionary dynamics between pollinators and microorganisms, and their potential ecological applications, to deepen our understanding of the complex interactions within plant ecosystems.

## 5 Conclusion

The findings of this study revealed that *C. Reticulata* nectar primarily relies on nectar antibacterial metabolites, especially H_2_O_2_, to regulate the population of nectar microorganisms and maintain nectar homeostasis. However, when the microorganisms overcome the physiological defense line of *C. Reticulata* nectar – namely the control exerted by bacteriostatic substances, their numbers can proliferate unchecked, leading to nectar spoilage and deterioration. Some pathogenic microorganisms can invade the nectarial tissues through the nectarthodes, causing flower tissue lesions, and disrupting the continuous secretion of substances that maintain nectar homeostasis through the nectarthodes. This imbalance in nectar ecology alters the color, odor, and chemical composition of nectar, hindering the mutual communication between *C. Reticulata* and pollinators, which ultimately affects the reproductive fitness of *C. Reticulata*. This study highlights the antagonistic effects of nectar antimicrobial metabolites on nectar bacteria, emphasizing the critical role of nectar homeostasis in supporting reproductive fitness in cross-pollinating plants.

## Data Availability

The original contributions presented in the study are included in the article/**Supplementary Material**. Further inquiries can be directed to the corresponding author.
