# Supplementary material for: Specialized metabolites present in Camellia reticulata nectar inhibit the growth of nectar‐inhabiting microorganisms
Source: Front Plant Sci. 2025 Mar 4;16:1557228. doi: 10.3389/fpls.2025.1557228 (PMC11913856; doi:10.3389/fpls.2025.1557228)
Supplement: Supplementary file 1 [file Table1.docx]

Supplementary Material

# Supplementary Table

**Supplementary Table 1.** The strains of microorganisms from *Camellia reticulata* nectar and their GenBank accession numbers

| Strains | Accession |
| --- | --- |
| Na-1 | PQ722026 |
| Na-2 | PQ722027 |
| Na-3 | PQ722028 |
| Na-4 | PQ722029 |
| Na-5 | PQ722030 |
| Na-6 | PQ722031 |
| S-1 | PQ722032 |
| S-2 | PQ722033 |
| S-3 | PQ722034 |
| S-4 | PQ722035 |
| S-5 | PQ722036 |
| S-6 | PQ722037 |
| S-7 | PQ722038 |
| S-8 | PQ722039 |
| S-9 | PQ722040 |
| S-10 | PQ722041 |
| S-11 | PQ722042 |
| S12 | PQ722043 |
| S13 | PQ722044 |
| Y-1 | PQ721930 |
| Y-2 | PQ721931 |

# Supplementary Data

LOCUS PQ722026 1364 bp DNA linear BCT 09-DEC-2024

DEFINITION Pseudomonas palleroniana strain Na-1 16S ribosomal RNA gene,

partial sequence.

ACCESSION PQ722026

VERSION PQ722026

KEYWORDS .

SOURCE Pseudomonas palleroniana

ORGANISM Pseudomonas palleroniana

Bacteria; Pseudomonadati; Pseudomonadota; Gammaproteobacteria;

Pseudomonadales; Pseudomonadaceae; Pseudomonas.

REFERENCE 1 (bases 1 to 1364)

AUTHORS Xun,L.

TITLE Direct Submission

JOURNAL Submitted (09-DEC-2024) College of Animal Science and Technology,

Yunnan Agricultural University, Fengyuan Road No. 452, Kunming,

Yunnan 650201, China

COMMENT ##Assembly-Data-START##

Sequencing Technology :: Sanger dideoxy sequencing

##Assembly-Data-END##

FEATURES Location/Qualifiers

source 1..1364

/organism="Pseudomonas palleroniana"

/mol_type="genomic DNA"

/strain="Na-1"

/db_xref="taxon:191390"

/geo_loc_name="China: Yunnan, Tengchong County"

/collection_date="16-Jun-2024"

rRNA <1..>1364

/product="16S ribosomal RNA"

ORIGIN

1 ttgcttctct tgagagcggc ggacgggtga gtaatgccta ggaatctgcc tggtagtggg

61 ggataacgtt cggaaacgga cgctaatacc gcatacgtcc tacgggagaa agcaggggac

121 cttcgggcct tgcgctatca gatgagccta ggtcggatta gctagttggt gaggtaatgg

181 ctcaccaagg cgacgatccg taactggtct gagaggatga tcagtcacac tggaactgag

241 acacggtcca gactcctacg ggaggcagca gtggggaata ttggacaatg ggcgaaagcc

301 tgatccagcc atgccgcgtg tgtgaagaag gtcttcggat tgtaaagcac tttaagttgg

361 gaggaagggc agttgcctaa tacgtaactg ttttgacgtt accgacagaa taagcaccgg

421 ctaactctgt gccagcagcc gcggtaatac agagggtgca agcgttaatc ggaattactg

481 ggcgtaaagc gcgcgtaggt ggtttgttaa gttggatgtg aaatccccgg gctcaacctg

541 ggaactgcat tcaaaactga ctgactagag tatggtagag ggtggtggaa tttcctgtgt

601 agcggtgaaa tgcgtagata taggaaggaa caccagtggc gaaggcgacc acctggactg

661 atactgacac tgaggtgcga aagcgtgggg agcaaacagg attagatacc ctggtagtcc

721 acgccgtaaa cgatgtcaac tagccgttgg aagccttgag cttttagtgg cgcagctaac

781 gcattaagtt gaccgcctgg ggagtacggc cgcaaggtta aaactcaaat gaattgacgg

841 gggcccgcac aagcggtgga gcatgtggtt taattcgaag caacgcgaag aaccttacca

901 ggccttgaca tccaatgaac tttccagaga tggattggtg ccttcgggaa cattgagaca

961 ggtgctgcat ggctgtcgtc agctcgtgtc gtgagatgtt gggttaagtc ccgtaacgag

1021 cgcaaccctt gtccttagtt accagcacgt tatggtgggc actctaagga gactgccggt

1081 gacaaaccgg aggaaggtgg ggatgacgtc aagtcatcat ggcccttacg gcctgggcta

1141 cacacgtgct acaatggtcg gtacaaaggg ttgccaagcc gcgaggtgga gctaatccca

1201 taaaaccgat cgtagtccgg atcgcagtct gcaactcgac tgcgtgaagt cggaatcgct

1261 agtaatcgcg aatcagaatg tcgcggtgaa tacgttcccg ggccttgtac acaccgcccg

1321 tcacaccatg ggagtgggtt gcaccagaag tagctagtct aacc

//

LOCUS PQ722027 1248 bp DNA linear BCT 09-DEC-2024

DEFINITION Rothia terrae strain Na-2 16S ribosomal RNA gene, partial sequence.

ACCESSION PQ722027

VERSION PQ722027

KEYWORDS .

SOURCE Rothia terrae

ORGANISM Rothia terrae

Bacteria; Bacillati; Actinomycetota; Actinomycetes; Micrococcales;

Micrococcaceae; Rothia.

REFERENCE 1 (bases 1 to 1248)

AUTHORS Xun,L.

TITLE Direct Submission

JOURNAL Submitted (09-DEC-2024) College of Animal Science and Technology,

Yunnan Agricultural University, Fengyuan Road No. 452, Kunming,

Yunnan 650201, China

COMMENT ##Assembly-Data-START##

Sequencing Technology :: Sanger dideoxy sequencing

##Assembly-Data-END##

FEATURES Location/Qualifiers

source 1..1248

/organism="Rothia terrae"

/mol_type="genomic DNA"

/strain="Na-2"

/db_xref="taxon:396015"

/geo_loc_name="China: Yunnan, Tengchong County"

/collection_date="16-Jun-2024"

rRNA <1..>1248

/product="16S ribosomal RNA"

ORIGIN

1 cctgcctttg actctgggat aagccttgga aacgaggtct aataccggat atgacacagt

61 ttcgcatgaa atctgtgtgg aaagggtttg tactggtttt agatgggctc acggcctatc

121 agcttgttgg tggggtaatg gctcaccaag gcgacgacgg gtagccggcc tgagagggtg

181 accggccaca ctgggactga gacacggccc agactcctac gggaggcagc agtggggaat

241 attgcacaat gggcgcaagc ctgatgcagc gacgccgcgt gagggatgac ggccttcggg

301 ttgtaaacct ctttcagcag gggagaagcg aaagtgacgg tacctgcaga agaagcgccg

361 gctaactacg tgccagcagc cgcggtaata cgtagggcgc gagcgttgtc cggaattatt

421 gggcgtaaag agcttgtagg cggtttgtcg cgtctgctgt gaaagcccgg ggcttaactc

481 cgggtttgca gtgggtacgg gctaactaga gtgcagtagg ggagactgga attcctggtg

541 tagcggtgga atgcgcagat atcaggagga acaccgatgg cgaaggcagg tctctgggct

601 gtaactgacg ctgagaagcg aaagcatggg gagcgaacag gattagatac cctggtagtc

661 catgccgtaa acgttgggca ctaggtgtgg gggacattcc acgttttccg cgccgtagct

721 aacgcattaa gtgccccgcc tggggagtac ggccgcaagg ctaaaactca aagaaattga

781 cgggggcccg cacaagcggc ggagcatgcg gattaattcg atgcaacgcg aagaacctta

841 ccaaggcttg acatatactg gactccccta gagatagggt ttcccttcgg ggctggtata

901 caggtggtgc atggttgtcg tcagctcgtg tcgtgagatg ttgggttaag tcccgcaacg

961 agcgcaaccc tcgttctatg ttgccagcac gtgatggtgg ggactcatag gagactgccg

1021 gggtcaactc ggaggaaggt ggggatgacg tcaaatcatc atgcccctta tgtcttgggc

1081 ttcacgcatg ctacaatggc cggtacaaag ggttgcgata ctgtgaggtt gagctaatcc

1141 caaaaagccg gtctcagttc ggattggggt ctgcaactcg accccatgaa gtcggagtcg

1201 ctagtaatcg cagatcagca acgctgcggt gaatacgttc ccgggcct

//

LOCUS PQ722028 1391 bp DNA linear BCT 09-DEC-2024

DEFINITION Bacillus subtilis strain Na-3 16S ribosomal RNA gene, partial

sequence.

ACCESSION PQ722028

VERSION PQ722028

KEYWORDS .

SOURCE Bacillus subtilis

ORGANISM Bacillus subtilis

Bacteria; Bacillati; Bacillota; Bacilli; Bacillales; Bacillaceae;

Bacillus.

REFERENCE 1 (bases 1 to 1391)

AUTHORS Xun,L.

TITLE Direct Submission

JOURNAL Submitted (09-DEC-2024) College of Animal Science and Technology,

Yunnan Agricultural University, Fengyuan Road No. 452, Kunming,

Yunnan 650201, China

COMMENT ##Assembly-Data-START##

Sequencing Technology :: Sanger dideoxy sequencing

##Assembly-Data-END##

FEATURES Location/Qualifiers

source 1..1391

/organism="Bacillus subtilis"

/mol_type="genomic DNA"

/strain="Na-3"

/db_xref="taxon:1423"

/geo_loc_name="China: Yunnan, Tengchong County"

/collection_date="16-Jun-2024"

rRNA <1..>1391

/product="16S ribosomal RNA"

ORIGIN

1 tgggagcttg ctccctgatg ttagcggcgg acgggtgagt aacacgtggg taacctgcct

61 gtaagactgg gataactccg ggaaaccggg gctaataccg gatggttgtt tgaaccgcat

121 ggttcaaaca taaaaggtgg ctttggctac cacttacaga tggacccgcg gcgcattagc

181 tagttggtga ggtaacggct caccaaggca acgatgcgta gccgacctga gagggtgatc

241 ggccacactg ggactgagac acggcccaga ctcctacggg aggcagcagt agggaatctt

301 ccgcaatgga cgaaagtctg acggagcaac gccgcgtgag tgatgaaggt tttcggatcg

361 taaagctctg ttgttaggga agaacaagta ccgttcgaat agggcggtac cttgacggta

421 cctaaccaga aagccacggc taactacgtg ccagcagccg cggtaatacg taggtggcaa

481 gcgttgtccg gaattattgg gcgtaaaggg ctcgcaggcg gtttcttaag tctgatgtga

541 aagcccccgg ctcaaccggg gagggtcatt ggaaactggg gaacttgagt gcagaagagg

601 agagtggaat tccacgtgta gcggtgaaat gcgtagagat gtggaggaac accagtggcg

661 aaggcgactc tctggtctgt aactgacgct gaggagcgaa agcgtgggga gcgaacagga

721 ttagataccc tggtagtcca cgccgtaaac gatgagtgct aagtgttagg gggtttccgc

781 cccttagtgc tgcagctaac gcattaagca ctccgcctgg ggagtacggt cgcaagactg

841 aaactcaaag gaattgacgg gggcccgcac aagcggtgga gcatgtggtt taattcgaag

901 caacgcgaag aaccttacca ggtcttgaca tcctctgaca atcctagaga taggacgtcc

961 ccttcggggg cagagtgaca ggtggtgcat ggttgtcgtc agctcgtgtc gtgagatgtt

1021 gggttaagtc ccgcaacgag cgcaaccctt gatcttagtt gccagcattc agttgggcac

1081 tctaaggtga ctgccggtga caaaccggag gaaggtgggg atgacgtcaa atcatcatgc

1141 cccttatgac ctgggctaca cacgtgctac aatggacaga acaaagggca gcgaaaccgc

1201 gaggttaagc caatcccaca aatctgttct cagttcggat cgcagtctgc aactcgactg

1261 cgtgaagctg gaatcgctag taatcgcgga tcagcatgcc gcggtgaata cgttcccggg

1321 ccttgtacac accgcccgtc acaccacgag agtttgtaac acccgaagtc ggtgaggtac

1381 ctttaggagc c

//

LOCUS PQ722029 1375 bp DNA linear BCT 09-DEC-2024

DEFINITION Rothia terrae strain Na-4 16S ribosomal RNA gene, partial sequence.

ACCESSION PQ722029

VERSION PQ722029

KEYWORDS .

SOURCE Rothia terrae

ORGANISM Rothia terrae

Bacteria; Bacillati; Actinomycetota; Actinomycetes; Micrococcales;

Micrococcaceae; Rothia.

REFERENCE 1 (bases 1 to 1375)

AUTHORS Xun,L.

TITLE Direct Submission

JOURNAL Submitted (09-DEC-2024) College of Animal Science and Technology,

Yunnan Agricultural University, Fengyuan Road No. 452, Kunming,

Yunnan 650201, China

COMMENT ##Assembly-Data-START##

Sequencing Technology :: Sanger dideoxy sequencing

##Assembly-Data-END##

FEATURES Location/Qualifiers

source 1..1375

/organism="Rothia terrae"

/mol_type="genomic DNA"

/strain="Na-4"

/db_xref="taxon:396015"

/geo_loc_name="China: Yunnan, Tengchong County"

/collection_date="16-Jun-2024"

rRNA <1..>1375

/product="16S ribosomal RNA"

ORIGIN

1 aagaccggtg cttgcactgg ttggattagt ggcgaacggg tgagtaatac gtgagtaacc

61 tgcctttgac tctgggataa gccttggaaa cgaggtctaa taccggatat gacacagttt

121 cgcatgaaat ctgtgtggaa agggtttgta ctggttttag atgggctcac ggcctatcag

181 cttgttggtg gggtaatggc tcaccaaggc gacgacgggt agccggcctg agagggtgac

241 cggccacact gggactgaga cacggcccag actcctacgg gaggcagcag tggggaatat

301 tgcacaatgg gcgcaagcct gatgcagcga cgccgcgtga gggatgacgg ccttcgggtt

361 gtaaacctct ttcagcaggg gagaagcgaa agtgacggta cctgcagaag aagcgccggc

421 taactacgtg ccagcagccg cggtaatacg tagggcgcga gcgttgtccg gaattattgg

481 gcgtaaagag cttgtaggcg gtttgtcgcg tctgctgtga aagcccgggg cttaactccg

541 ggtttgcagt gggtacgggc taactagagt gcagtagggg agactggaat tcctggtgta

601 gcggtggaat gcgcagatat caggaggaac accgatggcg aaggcaggtc tctgggctgt

661 aactgacgct gagaagcgaa agcatgggga gcgaacagga ttagataccc tggtagtcca

721 tgccgtaaac gttgggcact aggtgtgggg gacattccac gttttccgcg ccgtagctaa

781 cgcattaagt gccccgcctg gggagtacgg ccgcaaggct aaaactcaaa gaaattgacg

841 ggggcccgca caagcggcgg agcatgcgga ttaattcgat gcaacgcgaa gaaccttacc

901 aaggcttgac atatactgga ctcccctaga gatagggttt cccttcgggg ctggtataca

961 ggtggtgcat ggttgtcgtc agctcgtgtc gtgagatgtt gggttaagtc ccgcaacgag

1021 cgcaaccctc gttctatgtt gccagcacgt gatggtgggg actcatagga gactgccggg

1081 gtcaactcgg aggaaggtgg ggatgacgtc aaatcatcat gccccttatg tcttgggctt

1141 cacgcatgct acaatggccg gtacaaaggg ttgcgatact gtgaggttga gctaatccca

1201 aaaagccggt ctcagttcgg attggggtct gcaactcgac cccatgaagt cggagtcgct

1261 agtaatcgca gatcagcaac gctgcggtga atacgttccc gggccttgta cacaccgccc

1321 gtcaagtcac gaaagttggt aacacccgaa gccgatggcc taaccttttg gaggg

//

LOCUS PQ722030 1328 bp DNA linear BCT 09-DEC-2024

DEFINITION Agrobacterium rosae strain Na-5 16S ribosomal RNA gene, partial

sequence.

ACCESSION PQ722030

VERSION PQ722030

KEYWORDS .

SOURCE Agrobacterium rosae

ORGANISM Agrobacterium rosae

Bacteria; Pseudomonadati; Pseudomonadota; Alphaproteobacteria;

Hyphomicrobiales; Rhizobiaceae; Rhizobium/Agrobacterium group;

Agrobacterium.

REFERENCE 1 (bases 1 to 1328)

AUTHORS Xun,L.

TITLE Direct Submission

JOURNAL Submitted (09-DEC-2024) College of Animal Science and Technology,

Yunnan Agricultural University, Fengyuan Road No. 452, Kunming,

Yunnan 650201, China

COMMENT ##Assembly-Data-START##

Sequencing Technology :: Sanger dideoxy sequencing

##Assembly-Data-END##

FEATURES Location/Qualifiers

source 1..1328

/organism="Agrobacterium rosae"

/mol_type="genomic DNA"

/strain="Na-5"

/db_xref="taxon:1972867"

/geo_loc_name="China: Yunnan, Tengchong County"

/collection_date="16-Jun-2024"

rRNA <1..>1328

/product="16S ribosomal RNA"

ORIGIN

1 ggggagtggc agacgggtga gtaacgcgtg ggaatctacc catctctgcg gaatagctct

61 gggaaactgg aattaatacc gcatacgccc tacgggggaa agatttatcg gggatggatg

121 agcccgcgtt ggattagcta gttggtgggg taaaggccta ccaaggcgac gatccatagc

181 tggtctgaga ggatgatcag ccacattggg actgagacac ggcccaaact cctacgggag

241 gcagcagtgg ggaatattgg acaatgggcg caagcctgat ccagccatgc cgcgtgagtg

301 atgaaggcct tagggttgta aagctctttc accggtgaag ataatgacgg taaccggaga

361 agaagccccg gctaacttcg tgccagcagc cgcggtaata cgaagggggc tagcgttgtt

421 cggaattact gggcgtaaag cgcacgtagg cggatattta agtcaggggt gaaatcccag

481 agctcaactc tggaactgcc tttgatactg ggtatcttga gtatggaaga ggtaagtgga

541 attgcgagtg tagaggtgaa attcgtagat attcgcagga acaccagtgg cgaaggcggc

601 ttactggtcc attactgacg ctgaggtgcg aaagcgtggg gagcaaacag gattagatac

661 cctggtagtc cacgccgtaa acgatgaatg ttagccgtcg ggcagtctac tgttcggtgg

721 cgcagctaac gcattaaaca ttccgcctgg ggagtacggt cgcaagatta aaactcaaag

781 gaattgacgg gggcccgcac aagcggtgga gcatgtggtt taattcgaag caacgcgcag

841 aaccttacca gctcttgaca ttcggggttt gggcagtgga gacattgtcc ttcagttagg

901 ctggccccag aacaggtgct gcatggctgt cgtcagctcg tgtcgtgaga tgttgggtta

961 agtcccgcaa cgagcgcaac cctcgccctt agttgccagc atttggttgg gcactctaag

1021 gggactgccg gtgataagcc gagaggaagg tggggatgac gtcaagtcct catggccctt

1081 acgggctggg ctacacacgt gctacaatgg tggtgacagt gggcagcgag acagcgatgt

1141 cgagctaatc tccaaaagcc atctcagttc ggattgcact ctgcaactcg agtgcatgaa

1201 gttggaatcg ctagtaatcg cagatcagca tgctgcggtg aatacgttcc cgggccttgt

1261 acacaccgcc cgtcacacca tgggagttgg ttttacccga aggcgctgcg ctaaccgcaa

1321 gggggcag

//

LOCUS PQ722031 1375 bp DNA linear BCT 09-DEC-2024

DEFINITION Rothia sp. (in: high G+C Gram-positive bacteria) strain Na-6 16S

ribosomal RNA gene, partial sequence.

ACCESSION PQ722031

VERSION PQ722031

KEYWORDS .

SOURCE Rothia sp. (in: high G+C Gram-positive bacteria)

ORGANISM Rothia sp. (in: high G+C Gram-positive bacteria)

Bacteria; Bacillati; Actinomycetota; Actinomycetes; Micrococcales;

Micrococcaceae; Rothia.

REFERENCE 1 (bases 1 to 1375)

AUTHORS Xun,L.

TITLE Direct Submission

JOURNAL Submitted (09-DEC-2024) College of Animal Science and Technology,

Yunnan Agricultural University, Fengyuan Road No. 452, Kunming,

Yunnan 650201, China

COMMENT ##Assembly-Data-START##

Sequencing Technology :: Sanger dideoxy sequencing

##Assembly-Data-END##

FEATURES Location/Qualifiers

source 1..1375

/organism="Rothia sp. (in: high G+C Gram-positive

bacteria)"

/mol_type="genomic DNA"

/strain="Na-6"

/db_xref="taxon:1885016"

/geo_loc_name="China: Yunnan, Tengchong County"

/collection_date="16-Jun-2024"

rRNA <1..>1375

/product="16S ribosomal RNA"

ORIGIN

1 tcgaacgatg aagaccggtg cttgcactgg ttggattagt ggcgaacggg tgagtaatac

61 gtgagtaacc tgcctttgac tctgggataa gccttggaaa cgaggtctaa taccggatat

121 gacacagttt cgcatgaaat ctgtgtggaa agggtttgta ctggttttag atgggctcac

181 ggcctatcag cttgttggtg gggtaatggc tcaccaaggc gacgacgggt agccggcctg

241 agagggtgac cggccacact gggactgaga cacggcccag actcctacgg gaggcagcag

301 tggggaatat tgcacaatgg gcgcaagcct gatgcagcga cgccgcgtga gggatgacgg

361 ccttcgggtt gtaaacctct ttcagcaggg gagaagcgaa agtgacggta cctgcagaag

421 aagcgccggc taactacgtg ccagcagccg cggtaatacg tagggcgcga gcgttgtccg

481 gaattattgg gcgtaaagag cttgtaggcg gtttgtcgcg tctgctgtga aagcccgggg

541 cttaactccg ggtttgcagt gggtacgggc taactagagt gcagtagggg agactggaat

601 tcctggtgta gcggtggaat gcgcagatat caggaggaac accgatggcg aaggcaggtc

661 tctgggctgt aactgacgct gagaagcgaa agcatgggga gcgaacagga ttagataccc

721 tggtagtcca tgccgtaaac gttgggcact aggtgtgggg gacattccac gttttccgcg

781 ccgtagctaa cgcattaagt gccccgcctg gggagtacgg ccgcaaggct aaaactcaaa

841 gaaattgacg ggggcccgca caagcggcgg agcatgcgga ttaattcgat gcaacgcgaa

901 gaaccttacc aaggcttgac atatactgga ctcccctaga gatagggttt cccttcgggg

961 ctggtataca ggtggtgcat ggttgtcgtc agctcgtgtc gtgagatgtt gggttaagtc

1021 ccgcaacgag cgcaaccctc gttctatgtt gccagcacgt gatggtgggg actcatagga

1081 gactgccggg gtcaactcgg aggaaggtgg ggatgacgtc aaatcatcat gccccttatg

1141 tcttgggctt cacgcatgct acaatggccg gtacaaaggg ttgcgatact gtgaggttga

1201 gctaatccca aaaagccggt ctcagttcgg attggggtct gcaactcgac cccatgaagt

1261 cggagtcgct agtaatcgca gatcagcaac gctgcggtga atacgttccc gggccttgta

1321 cacaccgccc gtcaagtcac gaaagttggt aacacccgaa gccgatggcc taacc

//

LOCUS PQ722032 1387 bp DNA linear BCT 09-DEC-2024

DEFINITION Xanthomonas hydrangeae strain S-1 16S ribosomal RNA gene, partial

sequence.

ACCESSION PQ722032

VERSION PQ722032

KEYWORDS .

SOURCE Xanthomonas hydrangeae

ORGANISM Xanthomonas hydrangeae

Bacteria; Pseudomonadati; Pseudomonadota; Gammaproteobacteria;

Lysobacterales; Lysobacteraceae; Xanthomonas.

REFERENCE 1 (bases 1 to 1387)

AUTHORS Xun,L.

TITLE Direct Submission

JOURNAL Submitted (09-DEC-2024) College of Animal Science and Technology,

Yunnan Agricultural University, Fengyuan Road No. 452, Kunming,

Yunnan 650201, China

COMMENT ##Assembly-Data-START##

Sequencing Technology :: Sanger dideoxy sequencing

##Assembly-Data-END##

FEATURES Location/Qualifiers

source 1..1387

/organism="Xanthomonas hydrangeae"

/mol_type="genomic DNA"

/strain="S-1"

/db_xref="taxon:2775159"

/geo_loc_name="China: Yunnan, Tengchong County"

/collection_date="16-Jun-2024"

rRNA <1..>1387

/product="16S ribosomal RNA"

ORIGIN

1 aagagcttgc tcttatgggt ggcgagtggc ggacgggtga ggaatacatc ggaatctact

61 ctttcgtggg ggataacgta gggaaactta cgctaatacc gcatacgacc tacgggtgaa

121 agcggaggac cttcgggctt cgcgcgattg aatgagccga tgtcggatta gctagttggc

181 ggggtaaagg cccaccaagg cgacgatccg tagctggtct gagaggatga tcagccacac

241 tggaactgag acacggtcca gactcctacg ggaggcagca gtggggaata ttggacaatg

301 ggcgcaagcc tgatccagcc atgccgcgtg ggtgaagaag gccttcgggt tgtaaagccc

361 ttttgttggg aaagaaaagc agtcggttaa tacccgattg ttctgacggt acccaaagaa

421 taagcaccgg ctaacttcgt gccagcagcc gcggtaatac gaagggtgca agcgttactc

481 ggaattactg ggcgtaaagc gtgcgtaggt ggtggtttaa gtctgttgtg aaagccctgg

541 gctcaacctg ggaattgcag tggatactgg gtcactagag tgtggtagag ggtagcggaa

601 ttcccggtgt agcagtgaaa tgcgtagaga tcgggaggaa catccgtggc gaaggcggct

661 acctggacca acactgacac tgaggcacga aagcgtgggg agcaaacagg attagatacc

721 ctggtagtcc acgccctaaa cgatgcgaac tggatgttgg gtgcaatttg gcacgcagta

781 tcgaagctaa cgcgttaagt tcgccgcctg gggagtacgg tcgcaagact gaaactcaaa

841 ggaattgacg ggggcccgca caagcggtgg agtatgtggt ttaattcgat gcaacgcgaa

901 gaaccttacc tggtcttgac atccacggaa ctttccagag atggattggt gccttcggga

961 accgtgagac aggtgctgca tggctgtcgt cagctcgtgt cgtgagatgt tgggttaagt

1021 cccgcaacga gcgcaaccct tgtccttagt tgccagcacg taatggtggg aactctaagg

1081 agaccgccgg tgacaaaccg gaggaaggtg gggatgacgt caagtcatca tggcccttac

1141 gaccagggct acacacgtac tacaatggta gggacagagg gctgcaaacc cgcgagggta

1201 agccaatccc agaaacccta tctcagtccg gattggagtc tgcaactcga ctccatgaag

1261 tcggaatcgc tagtaatcgc agatcagcat tgctgcggtg aatacgttcc cgggccttgt

1321 acacaccgcc cgtcacacca tgggagtttg ttgcaccaga agcaggtagc ttaaccttcg

1381 ggagggc

//

LOCUS PQ722033 1364 bp DNA linear BCT 09-DEC-2024

DEFINITION Pantoea agglomerans strain S-2 16S ribosomal RNA gene, partial

sequence.

ACCESSION PQ722033

VERSION PQ722033

KEYWORDS .

SOURCE Pantoea agglomerans

ORGANISM Pantoea agglomerans

Bacteria; Pseudomonadati; Pseudomonadota; Gammaproteobacteria;

Enterobacterales; Erwiniaceae; Pantoea; Pantoea agglomerans group.

REFERENCE 1 (bases 1 to 1364)

AUTHORS Xun,L.

TITLE Direct Submission

JOURNAL Submitted (09-DEC-2024) College of Animal Science and Technology,

Yunnan Agricultural University, Fengyuan Road No. 452, Kunming,

Yunnan 650201, China

COMMENT ##Assembly-Data-START##

Sequencing Technology :: Sanger dideoxy sequencing

##Assembly-Data-END##

FEATURES Location/Qualifiers

source 1..1364

/organism="Pantoea agglomerans"

/mol_type="genomic DNA"

/strain="S-2"

/db_xref="taxon:549"

/geo_loc_name="China: Yunnan, Tengchong County"

/collection_date="16-Jun-2024"

rRNA <1..>1364

/product="16S ribosomal RNA"

ORIGIN

1 tgctcttggg tgacgagtgg cggacgggtg agtaatgtct ggggatctgc ccgatagagg

61 gggataacca ctggaaacgg tggctaatac cgcataacgt cgcaagacca aagaggggga

121 ccttcgggcc tctcactatc ggatgaaccc agatgggatt agctagtagg cggggtaatg

181 gcccacctag gcgacgatcc ctagctggtc tgagaggatg accagccaca ctggaactga

241 gacacggtcc agactcctac gggaggcagc agtggggaat attgcacaat gggcgcaagc

301 ctgatgcagc catgccgcgt gtatgaagaa ggccttcggg ttgtaaagta ctttcagcgg

361 ggaggaaggc gacgcggtta ataaccgtgt cgattgacgt tacccgcaga agaagcaccg

421 gctaactccg tgccagcagc cgcggtaata cggagggtgc aagcgttaat cggaattact

481 gggcgtaaag cgcacgcagg cggtctgtta agtcagatgt gaaatccccg ggcttaacct

541 gggaactgca tttgaaactg gcaggcttga gtcttgtaga ggggggtaga attccaggtg

601 tagcggtgaa atgcgtagag atctggagga ataccggtgg cgaaggcggc cccctggaca

661 aagactgacg ctcaggtgcg aaagcgtggg gagcaaacag gattagatac cctggtagtc

721 cacgccgtaa acgatgtcga cttggaggtt gttcccttga ggagtggctt ccggagctaa

781 cgcgttaagt cgaccgcctg gggagtacgg ccgcaaggtt aaaactcaaa tgaattgacg

841 ggggcccgca caagcggtgg agcatgtggt ttaattcgat gcaacgcgaa gaaccttacc

901 tactcttgac atccagcgaa ttcggcagag atgccttagt gccttcggga acgctgagac

961 aggtgctgca tggctgtcgt cagctcgtgt tgtgaaatgt tgggttaagt cccgcaacga

1021 gcgcaaccct tatcctttgt tgccagcgcg tcatggtggg aactcaaagg agactgccgg

1081 tgataaaccg gaggaaggtg gggatgacgt caagtcatca tggcccttac gagtagggct

1141 acacacgtgc tacaatggcg catacaaaga gaagcgacct cgcgagagca agcggacctc

1201 acaaagtgcg tcgtagtccg gatcggagtc tgcaactcga ctccgtgaag tcggaatcgc

1261 tagtaatcgt ggatcagaat gccacggtga atacgttccc gggccttgta cacaccgccc

1321 gtcacaccat gggagtgggt tgcaaaagaa gtaggtagct taac

//

LOCUS PQ722034 1338 bp DNA linear BCT 09-DEC-2024

DEFINITION Rothia terrae strain S-3 16S ribosomal RNA gene, partial sequence.

ACCESSION PQ722034

VERSION PQ722034

KEYWORDS .

SOURCE Rothia terrae

ORGANISM Rothia terrae

Bacteria; Bacillati; Actinomycetota; Actinomycetes; Micrococcales;

Micrococcaceae; Rothia.

REFERENCE 1 (bases 1 to 1338)

AUTHORS Xun,L.

TITLE Direct Submission

JOURNAL Submitted (09-DEC-2024) College of Animal Science and Technology,

Yunnan Agricultural University, Fengyuan Road No. 452, Kunming,

Yunnan 650201, China

COMMENT ##Assembly-Data-START##

Sequencing Technology :: Sanger dideoxy sequencing

##Assembly-Data-END##

FEATURES Location/Qualifiers

source 1..1338

/organism="Rothia terrae"

/mol_type="genomic DNA"

/strain="S-3"

/db_xref="taxon:396015"

/geo_loc_name="China: Yunnan, Tengchong County"

/collection_date="16-Jun-2024"

rRNA <1..>1338

/product="16S ribosomal RNA"

ORIGIN

1 tggttggatt agtggcgaac gggtgagtaa tacgtgagta acctgccttt gactctggga

61 taagccttgg aaacgaggtc taataccgga tatgacacag tttcgcatga aatctgtgtg

121 gaaagggttt gtactggttt tagatgggct cacggcctat cagcttgttg gtggggtaat

181 ggctcaccaa ggcgacgacg ggtagccggc ctgagagggt gaccggccac actgggactg

241 agacacggcc cagactccta cgggaggcag cagtggggaa tattgcacaa tgggcgcaag

301 cctgatgcag cgacgccgcg tgagggatga cggccttcgg gttgtaaacc tctttcagca

361 ggggagaagc gaaagtgacg gtacctgcag aagaagcgcc ggctaactac gtgccagcag

421 ccgcggtaat acgtagggcg cgagcgttgt ccggaattat tgggcgtaaa gagcttgtag

481 gcggtttgtc gcgtctgctg tgaaagcccg gggcttaact ccgggtttgc agtgggtacg

541 ggctaactag agtgcagtag gggagactgg aattcctggt gtagcggtgg aatgcgcaga

601 tatcaggagg aacaccgatg gcgaaggcag gtctctgggc tgtaactgac gctgagaagc

661 gaaagcatgg ggagcgaaca ggattagata ccctggtagt ccatgccgta aacgttgggc

721 actaggtgtg ggggacattc cacgttttcc gcgccgtagc taacgcatta agtgccccgc

781 ctggggagta cggccgcaag gctaaaactc aaagaaattg acgggggccc gcacaagcgg

841 cggagcatgc ggattaattc gatgcaacgc gaagaacctt accaaggctt gacatatact

901 ggactcccct agagataggg tttcccttcg gggctggtat acaggtggtg catggttgtc

961 gtcagctcgt gtcgtgagat gttgggttaa gtcccgcaac gagcgcaacc ctcgttctat

1021 gttgccagca cgtgatggtg gggactcata ggagactgcc ggggtcaact cggaggaagg

1081 tggggatgac gtcaaatcat catgcccctt atgtcttggg cttcacgcat gctacaatgg

1141 ccggtacaaa gggttgcgat actgtgaggt tgagctaatc ccaaaaagcc ggtctcagtt

1201 cggattgggg tctgcaactc gaccccatga agtcggagtc gctagtaatc gcagatcagc

1261 aacgctgcgg tgaatacgtt cccgggcctt gtacacaccg cccgtcaagt cacgaaagtt

1321 ggtaacaccc gaagccga

//

LOCUS PQ722035 1399 bp DNA linear BCT 09-DEC-2024

DEFINITION Lelliottia amnigena strain S-4 16S ribosomal RNA gene, partial

sequence.

ACCESSION PQ722035

VERSION PQ722035

KEYWORDS .

SOURCE Lelliottia amnigena

ORGANISM Lelliottia amnigena

Bacteria; Pseudomonadati; Pseudomonadota; Gammaproteobacteria;

Enterobacterales; Enterobacteriaceae; Lelliottia.

REFERENCE 1 (bases 1 to 1399)

AUTHORS Xun,L.

TITLE Direct Submission

JOURNAL Submitted (09-DEC-2024) College of Animal Science and Technology,

Yunnan Agricultural University, Fengyuan Road No. 452, Kunming,

Yunnan 650201, China

COMMENT ##Assembly-Data-START##

Sequencing Technology :: Sanger dideoxy sequencing

##Assembly-Data-END##

FEATURES Location/Qualifiers

source 1..1399

/organism="Lelliottia amnigena"

/mol_type="genomic DNA"

/strain="S-4"

/db_xref="taxon:61646"

/geo_loc_name="China: Yunnan, Tengchong County"

/collection_date="16-Jun-2024"

rRNA <1..>1399

/product="16S ribosomal RNA"

ORIGIN

1 agtcgagcgg tagccagaga gcttgctctc gggtgacgag cggcggacgg gtgagtaatg

61 tctgggaaac tgcctgatgg agggggataa ctactggaaa cggtagctaa taccgcataa

121 cgtcgcaaga ccaaagaggg ggaccttcgg gcctcttgcc atcagatgtg cccagatggg

181 attagctagt aggtggggta atggctcacc taggcgacga tccctagctg gtctgagagg

241 atgaccagcc acactggaac tgagacacgg tccagactcc tacgggaggc agcagtgggg

301 aatattgcac aatgggcgca agcctgatgc agccatgccg cgtgtatgaa gaaggccttc

361 gggttgtaaa gtactttcag cgaggaggaa ggcattgtgg ttaataaccg cagtgattga

421 cgttactcgc agaagaagca ccggctaact ccgtgccagc agccgcggta atacggaggg

481 tgcaagcgtt aatcggaatt actgggcgta aagcgcacgc aggcggtctg tcaagtcgga

541 tgtgaaatcc ccgggctcaa cctgggaact gcattcgaaa ctggcaggct agagtcttgt

601 agaggggggt agaattccag gtgtagcggt gaaatgcgta gagatctgga ggaataccgg

661 tggcgaaggc ggccccctgg acaaagactg acgctcaggt gcgaaagcgt ggggagcaaa

721 caggattaga taccctggta gtccacgccg taaacgatgt cgacttggag gttgttccct

781 tgaggagtgg cttccggagc taacgcgtta agtcgaccgc ctggggagta cggccgcaag

841 gttaaaactc aaatgaattg acgggggccc gcacaagcgg tggagcatgt ggtttaattc

901 gatgcaacgc gaagaacctt acctactctt gacatccaga gaacttagca gagatgcttt

961 ggtgccttcg ggaactctga gacaggtgct gcatggctgt cgtcagctcg tgttgtgaaa

1021 tgttgggtta agtcccgcaa cgagcgcaac ccttatcctt tgttgccagc ggttcggccg

1081 ggaactcaaa ggagactgcc agtgataaac tggaggaagg tggggatgac gtcaagtcat

1141 catggccctt acgagtaggg ctacacacgt gctacaatgg catatacaaa gagaagcgac

1201 ctcgcgagag caagcggacc tcacaaagta tgtcgtagtc cggatcggag tctgcaactc

1261 gactccgtga agtcggaatc gctagtaatc gtagatcaga atgctacggt gaatacgttc

1321 ccgggccttg tacacaccgc ccgtcacacc atgggagtgg gttgcaaaag aagtaggtag

1381 cttaaccttc gggagggcg

//

LOCUS PQ722036 1370 bp DNA linear BCT 09-DEC-2024

DEFINITION Curtobacterium flaccumfaciens strain S-5 16S ribosomal RNA gene,

partial sequence.

ACCESSION PQ722036

VERSION PQ722036

KEYWORDS .

SOURCE Curtobacterium flaccumfaciens

ORGANISM Curtobacterium flaccumfaciens

Bacteria; Bacillati; Actinomycetota; Actinomycetes; Micrococcales;

Microbacteriaceae; Curtobacterium.

REFERENCE 1 (bases 1 to 1370)

AUTHORS Xun,L.

TITLE Direct Submission

JOURNAL Submitted (09-DEC-2024) College of Animal Science and Technology,

Yunnan Agricultural University, Fengyuan Road No. 452, Kunming,

Yunnan 650201, China

COMMENT ##Assembly-Data-START##

Sequencing Technology :: Sanger dideoxy sequencing

##Assembly-Data-END##

FEATURES Location/Qualifiers

source 1..1370

/organism="Curtobacterium flaccumfaciens"

/mol_type="genomic DNA"

/strain="S-5"

/db_xref="taxon:2035"

/geo_loc_name="China: Yunnan, Tengchong County"

/collection_date="16-Jun-2024"

rRNA <1..>1370

/product="16S ribosomal RNA"

ORIGIN

1 cagtcgaacg atgatcagga gcttgctctt gtgattagtg gcgaacgggt gagtaacacg

61 tgagtaacct gcccctgact ctgggataag cgttggaaac gacgtctaat actggatatg

121 acttccggcc gcatggtctg gttgtggaaa gattttttgg ttggggatgg actcgcggcc

181 tatcagcttg ttggtgaggt aatggctcac caaggcgacg acgggtagcc ggcctgagag

241 ggtgaccggc cacactggga ctgagacacg gcccagactc ctacgggagg cagcagtggg

301 gaatattgca caatgggcga aagcctgatg cagcaacgcc gcgtgaggga tgacggcctt

361 cgggttgtaa acctctttta gtagggaaga agcgaaagtg acggtacctg cagaaaaagc

421 accggctaac tacgtgccag cagccgcggt aatacgtagg gtgcaagcgt tgtccggaat

481 tattgggcgt aaagagctcg taggcggttt gtcgcgtctg ctgtgaaatc ccgaggctca

541 acctcgggct tgcagtgggt acgggcagac tagagtgcgg taggggagat tggaattcct

601 ggtgtagcgg tggaatgcgc agatatcagg aggaacaccg atggcgaagg cagatctctg

661 ggccgtaact gacgctgagg agcgaaagca tggggagcga acaggattag ataccctggt

721 agtccatgcc gtaaacgttg ggcgctagat gtagggacct ttccacggtt tctgtgtcgt

781 agctaacgca ttaagcgccc cgcctgggga gtacggccgc aaggctaaaa ctcaaaggaa

841 ttgacggggg cccgcacaag cggcggagca tgcggattaa ttcgatgcaa cgcgaagaac

901 cttaccaagg cttgacatac accggaaacg gccagagatg gtcgccccct tgtggtcggt

961 gtacaggtgg tgcatggttg tcgtcagctc gtgtcgtgag atgttgggtt aagtcccgca

1021 acgagcgcaa ccctcgttct atgttgccag cgggttatgc cggggactca taggagactg

1081 ccggggtcaa ctcggaggaa ggtggggatg acgtcaaatc atcatgcccc ttatgtcttg

1141 ggcttcacgc atgctacaat ggccggtaca aagggctgcg ataccgtaag gtggagcgaa

1201 tcccaaaaag ccggtctcag ttcggattga ggtctgcaac tcgacctcat gaagtcggag

1261 tcgctagtaa tcgcagatca gcaacgctgc ggtgaatacg ttcccgggcc ttgtacacac

1321 cgcccgtcaa gtcatgaaag tcggtaacac ccgaagccgg tggcctaacc

//

LOCUS PQ722037 1406 bp DNA linear BCT 09-DEC-2024

DEFINITION Xanthomonas arboricola pv. pruni strain S-6 16S ribosomal RNA gene,

partial sequence.

ACCESSION PQ722037

VERSION PQ722037

KEYWORDS .

SOURCE Xanthomonas arboricola pv. pruni

ORGANISM Xanthomonas arboricola pv. pruni

Bacteria; Pseudomonadati; Pseudomonadota; Gammaproteobacteria;

Lysobacterales; Lysobacteraceae; Xanthomonas.

REFERENCE 1 (bases 1 to 1406)

AUTHORS Xun,L.

TITLE Direct Submission

JOURNAL Submitted (09-DEC-2024) College of Animal Science and Technology,

Yunnan Agricultural University, Fengyuan Road No. 452, Kunming,

Yunnan 650201, China

COMMENT ##Assembly-Data-START##

Sequencing Technology :: Sanger dideoxy sequencing

##Assembly-Data-END##

FEATURES Location/Qualifiers

source 1..1406

/organism="Xanthomonas arboricola pv. pruni"

/mol_type="genomic DNA"

/strain="S-6"

/db_xref="taxon:69929"

/geo_loc_name="China: Yunnan, Tengchong County"

/collection_date="16-Jun-2024"

/note="pathovar: pruni"

rRNA <1..>1406

/product="16S ribosomal RNA"

ORIGIN

1 cagtcgaacg gcagccagta agagcttgct cttatgggtg gcgagtggcg gacgggtgag

61 gaatacatcg gaatctactc tttcgtgggg gataacgtag ggaaacttac gctaataccg

121 catacgacct acgggtgaaa gcggaggacc ttcgggcttc gcgcgattga atgagccgat

181 gtcggattag ctagttggcg gggtaaaggc ccaccaaggc gacgatccgt agctggtctg

241 agaggatgat cagccacact ggaactgaga cacggtccag actcctacgg gaggcagcag

301 tggggaatat tggacaatgg gcgcaagcct gatccagcca tgccgcgtgg gtgaagaagg

361 ccttcgggtt gtaaagccct tttgttggga aagaaaagca gtcggttaat acccgattgt

421 tctgacggta cccaaagaat aagcaccggc taacttcgtg ccagcagccg cggtaatacg

481 aagggtgcaa gcgttactcg gaattactgg gcgtaaagcg tgcgtaggtg gtggtttaag

541 tctgttgtga aagccctggg ctcaacctgg gaattgcagt ggatactggg tcactagagt

601 gtggtagagg gtagcggaat tcccggtgta gcagtgaaat gcgtagagat cgggaggaac

661 atccgtggcg aaggcggcta cctggaccaa cactgacact gaggcacgaa agcgtgggga

721 gcaaacagga ttagataccc tggtagtcca cgccctaaac gatgcgaact ggatgttggg

781 tgcaatttgg cacgcagtat cgaagctaac gcgttaagtt cgccgcctgg ggagtacggt

841 cgcaagactg aaactcaaag gaattgacgg gggcccgcac aagcggtgga gtatgtggtt

901 taattcgatg caacgcgaag aaccttacct ggtcttgaca tccacggaac tttccagaga

961 tggattggtg ccttcgggaa ccgtgagaca ggtgctgcat ggctgtcgtc agctcgtgtc

1021 gtgagatgtt gggttaagtc ccgcaacgag cgcaaccctt gtccttagtt gccagcacgt

1081 aatggtggga actctaagga gaccgccggt gacaaaccgg aggaaggtgg ggatgacgtc

1141 aagtcatcat ggcccttacg accagggcta cacacgtact acaatggtag ggacagaggg

1201 ctgcaaaccc gcgagggtaa gccaatccca gaaaccctat ctcagtccgg attggagtct

1261 gcaactcgac tccatgaagt cggaatcgct agtaatcgca gatcagcatt gctgcggtga

1321 atacgttccc gggccttgta cacaccgccc gtcacaccat gggagtttgt tgcaccagaa

1381 gcaggtagct taacctcggg agggcg

//

LOCUS PQ722038 1399 bp DNA linear BCT 09-DEC-2024

DEFINITION Erwinia amylovora strain S-7 16S ribosomal RNA gene, partial

sequence.

ACCESSION PQ722038

VERSION PQ722038

KEYWORDS .

SOURCE Erwinia amylovora

ORGANISM Erwinia amylovora

Bacteria; Pseudomonadati; Pseudomonadota; Gammaproteobacteria;

Enterobacterales; Erwiniaceae; Erwinia.

REFERENCE 1 (bases 1 to 1399)

AUTHORS Xun,L.

TITLE Direct Submission

JOURNAL Submitted (09-DEC-2024) College of Animal Science and Technology,

Yunnan Agricultural University, Fengyuan Road No. 452, Kunming,

Yunnan 650201, China

COMMENT ##Assembly-Data-START##

Sequencing Technology :: Sanger dideoxy sequencing

##Assembly-Data-END##

FEATURES Location/Qualifiers

source 1..1399

/organism="Erwinia amylovora"

/mol_type="genomic DNA"

/strain="S-7"

/db_xref="taxon:552"

/geo_loc_name="China: Yunnan, Tengchong County"

/collection_date="16-Jun-2024"

rRNA <1..>1399

/product="16S ribosomal RNA"

ORIGIN

1 cagtcgaacg gtagccagag agcttgctct tgggtgacga gtggcggacg ggtgagtaat

61 gtctgggaaa ctgcccgatg gagggggata actactggaa acggtagcta ataccgcata

121 acgtcttcgg accaaagtgg gggaccttcg ggcctcacac catcggatgt gcccagatgg

181 gattagctag taggtggggt aatggctcac ctaggcgacg atccctagct ggtctgagag

241 gatgaccagc cacactggaa ctgagacacg gtccagactc ctacgggagg cagcagtggg

301 gaatattgca caatgggcgc aagcctgatg cagccatgcc gcgtgtatga agaaggcctt

361 cgggttgtaa agtactttca gcggggagga aggcgatgag gttaatagcc ttgtcgattg

421 acgttacccg cagaagaagc accggctaac tccgtgccag cagccgcggt aatacggagg

481 gtgcaagcgt taatcggaat tactgggcgt aaagcgcacg caggcggtct gtcaagtcgg

541 atgtgaaatc cccgggctca acccgggaac tgcattcgaa actggcagac tagagtctcg

601 tagagggggg tagaattcca ggtgtagcgg tgaaatgcgt agagatctgg aggaataccg

661 gtggcgaagg cggccccctg gacgaagact gacgctcagg tgcgaaagcg tggggagcaa

721 acaggattag ataccctggt agtccacgcc gtaaacgatg tcgacttgga ggctgttccc

781 ttgaggagtg gcttccggag ctaacgcgtt aagtcgaccg cctggggagt acggccgcaa

841 ggttaaaact caaatgaatt gacgggggcc cgcacaagcg gtggagcatg tggtttaatt

901 cgatgcaacg cgaagaacct tacctggcct tgacatccac ggaattctgc agagatgcgg

961 aagtgccttc gggaaccgtg agacaggtgc tgcatggctg tcgtcagctc gtgttgtgaa

1021 atgttgggtt aagtcccgca acgagcgcaa cccttatcct ttgttgccag cgattcggtc

1081 gggaactcaa aggagactgc cggtgataaa ccggaggaag gtggggatga cgtcaagtca

1141 tcatggccct tacggccagg gctacacacg tgctacaatg gcgcatacaa agagaagcga

1201 cctcgcgaga gcaagcggac ctcataaagt gcgtcgtagt ccggatcgga gtctgcaact

1261 cgactccgtg aagtcggaat cgctagtaat cgtagatcag aatgctacgg tgaatacgtt

1321 cccgggcctt gtacacaccg cccgtcacac catgggagtg ggttgcaaaa gaagtaggta

1381 gcttaacctt cgggagggc

//

LOCUS PQ722039 1374 bp DNA linear BCT 09-DEC-2024

DEFINITION Rosenbergiella epipactidis strain S-8 16S ribosomal RNA gene,

partial sequence.

ACCESSION PQ722039

VERSION PQ722039

KEYWORDS .

SOURCE Rosenbergiella epipactidis

ORGANISM Rosenbergiella epipactidis

Bacteria; Pseudomonadati; Pseudomonadota; Gammaproteobacteria;

Enterobacterales; Erwiniaceae; Rosenbergiella.

REFERENCE 1 (bases 1 to 1374)

AUTHORS Xun,L.

TITLE Direct Submission

JOURNAL Submitted (09-DEC-2024) College of Animal Science and Technology,

Yunnan Agricultural University, Fengyuan Road No. 452, Kunming,

Yunnan 650201, China

COMMENT ##Assembly-Data-START##

Sequencing Technology :: Sanger dideoxy sequencing

##Assembly-Data-END##

FEATURES Location/Qualifiers

source 1..1374

/organism="Rosenbergiella epipactidis"

/mol_type="genomic DNA"

/strain="S-8"

/db_xref="taxon:1544694"

/geo_loc_name="China: Yunnan, Tengchong County"

/collection_date="16-Jun-2024"

rRNA <1..>1374

/product="16S ribosomal RNA"

ORIGIN

1 gagagtagct tgctactttg ctgacgagtg gcggacgggt gagtaatgtc tggggatctg

61 cctgatggag ggggataact actggaaacg gtagctaata ccgcataacg tcgcaagacc

121 aaagcggggg actttcgagc ctcgcaccat cagatgaacc cagatgggat tagctagtag

181 gtaaggtaat ggcttaccta ggcgacgatc cctagctggt ctgagaggat gaccagccac

241 actggaactg agacacggtc cagactccta cgggaggcag cagtggggaa tattgcacaa

301 tgggcgcaag cctgatgcag ccatgccgcg tgtatgaaga aggccttcgg gttgtaaagt

361 actttcagtc aggaggaagg gtgtgaaatt aatactttca cgcattgacg ttactgacag

421 aagaagcacc ggctaactcc gtgccagcag ccgcggtaat acggagggtg caagcgttaa

481 tcggaattac tgggcgtaaa gcgcacgcag gcggtttgtt aagtcagatg tgaaatcccc

541 gggctcaacc tgggaactgc atttgaaact ggcaagcttg agtcttgtag aggggggtag

601 aattccaggt gtagcggtga aatgcgtaga gatctggagg aataccggtg gcgaaggcgg

661 ccccctggac aaagactgac gctcaggtgc gaaagcgtgg ggagcaaaca ggattagata

721 ccctggtagt ccacgccgta aacgatgtcg acttggaggc tgttcccttg aggagtggct

781 tccggagcta acgcgttaag tcgaccgcct ggggagtacg gccgcaaggt taaaactcaa

841 atgaattgac gggggcccgc acaagcggtg gagcatgtgg tttaattcga tgcaacgcga

901 agaaccttac ctactcttga catccagaga atttagcaga gatgccttag tgccttcggg

961 aactctgaga caggtgctgc atggctgtcg tcagctcgtg ttgtgaaatg ttgggttaag

1021 tcccgcaacg agcgcaaccc ttatcctttg ttgccagcac gtaatggtgg gaactcaaag

1081 gagactgccg gtgataaacc ggaggaaggt ggggatgacg tcaagtcatc atggccctta

1141 cgagtagggc tacacacgtg ctacaatggc gcatacaaag agaagcaaac tcgcgagagc

1201 aagcggacct cataaagtgc gtcgtagtcc ggattggagt ctgcaactcg actccatgaa

1261 gtcggaatcg ctagtaatcg tgaatcagaa tgtcacggtg aatacgttcc cgggccttgt

1321 acacaccgcc cgtcacacca tgggagtggg ttgcaaaaga agtagatagc ttaa

//

LOCUS PQ722040 1381 bp DNA linear BCT 09-DEC-2024

DEFINITION Leuconostoc suionicum strain S-9 16S ribosomal RNA gene, partial

sequence.

ACCESSION PQ722040

VERSION PQ722040

KEYWORDS .

SOURCE Leuconostoc suionicum

ORGANISM Leuconostoc suionicum

Bacteria; Bacillati; Bacillota; Bacilli; Lactobacillales;

Lactobacillaceae; Leuconostoc.

REFERENCE 1 (bases 1 to 1381)

AUTHORS Xun,L.

TITLE Direct Submission

JOURNAL Submitted (09-DEC-2024) College of Animal Science and Technology,

Yunnan Agricultural University, Fengyuan Road No. 452, Kunming,

Yunnan 650201, China

COMMENT ##Assembly-Data-START##

Sequencing Technology :: Sanger dideoxy sequencing

##Assembly-Data-END##

FEATURES Location/Qualifiers

source 1..1381

/organism="Leuconostoc suionicum"

/mol_type="genomic DNA"

/strain="S-9"

/db_xref="taxon:1511761"

/geo_loc_name="China: Yunnan, Tengchong County"

/collection_date="16-Jun-2024"

rRNA <1..>1381

/product="16S ribosomal RNA"

ORIGIN

1 gcgaaaggtg cttgcacctt tcaagtgagt ggcgaacggg tgagtaacac gtggacaacc

61 tgcctcaagg ctggggataa catttggaaa cagatgctaa taccgaataa aacttagtgt

121 cgcatgacac aaagttaaaa ggcgcttcgg cgtcacctag agatggatcc gcggtgcatt

181 agttagttgg tggggtaaag gcctaccaag acaatgatgc atagccgagt tgagagactg

241 atcggccaca ttgggactga gacacggccc aaactcctac gggaggctgc agtagggaat

301 cttccacaat gggcgaaagc ctgatggagc aacgccgcgt gtgtgatgaa ggctttcggg

361 tcgtaaagca ctgttgtatg ggaagaacag ctagaatagg aaatgatttt agtttgacgg

421 taccatacca gaaagggacg gctaaatacg tgccagcagc cgcggtaata cgtatgtccc

481 gagcgttatc cggatttatt gggcgtaaag cgagcgcaga cggtttatta agtctgatgt

541 gaaagcccgg agctcaactc cggaattgca ttggaaactg gttaacttga gtgcagtaga

601 ggtaagtgga actccatgtg tagcggtgga atgcgtagat atatggaaga acaccagtgg

661 cgaaggcggc ttactggact gcaactgacg ttgaggctcg aaagtgtggg tagcaaacag

721 gattagatac cctggtagtc cacaccgtaa acgatgaaca ctaggtgtta ggaggtttcc

781 gcctcttagt gccgaagcta acgcattaag tgttccgcct ggggagtacg accgcaaggt

841 tgaaactcaa aggaattgac ggggacccgc acaagcggtg gagcatgtgg tttaattcga

901 agcaacgcga agaaccttac caggtcttga catcctttga agcttttaga gatagaagtg

961 ttctcttcgg agacaaagtg acaggtggtg catggtcgtc gtcagctcgt gtcgtgagat

1021 gttgggttaa gtcccgcaac gagcgcaacc cttattgtta gttgccagca ttcagatggg

1081 cactctagcg agactgccgg tgacaaaccg gaggaaggcg gggacgacgt cagatcatca

1141 tgccccttat gacctgggct acacacgtgc tacaatggcg tatacaacga gttgccagcc

1201 cgcgagggtg agctaatctc ttaaagtacg tctcagttcg gattgtagtc tgcaactcga

1261 ctacatgaag tcggaatcgc tagtaatcgc ggatcagcac gccgcggtga atacgttccc

1321 gggtcttgta cacaccgccc gtcacaccat gggagtttgt aatgcccaaa gccggtggcc

1381 t

//

LOCUS PQ722041 1406 bp DNA linear BCT 09-DEC-2024

DEFINITION Xanthomonas campestris pv. raphani strain S-10 16S ribosomal RNA

gene, partial sequence.

ACCESSION PQ722041

VERSION PQ722041

KEYWORDS .

SOURCE Xanthomonas campestris pv. raphani

ORGANISM Xanthomonas campestris pv. raphani

Bacteria; Pseudomonadati; Pseudomonadota; Gammaproteobacteria;

Lysobacterales; Lysobacteraceae; Xanthomonas.

REFERENCE 1 (bases 1 to 1406)

AUTHORS Xun,L.

TITLE Direct Submission

JOURNAL Submitted (09-DEC-2024) College of Animal Science and Technology,

Yunnan Agricultural University, Fengyuan Road No. 452, Kunming,

Yunnan 650201, China

COMMENT ##Assembly-Data-START##

Sequencing Technology :: Sanger dideoxy sequencing

##Assembly-Data-END##

FEATURES Location/Qualifiers

source 1..1406

/organism="Xanthomonas campestris pv. raphani"

/mol_type="genomic DNA"

/strain="S-10"

/db_xref="taxon:359385"

/geo_loc_name="China: Yunnan, Tengchong County"

/collection_date="16-Jun-2024"

/note="pathovar: raphani"

rRNA <1..>1406

/product="16S ribosomal RNA"

ORIGIN

1 gtcgaacggc agccagtaag agcttgctct tatgggtggc gagtggcgga cgggtgagga

61 atacatcgga atctactctt tcgtggggga taacgtaggg aaacttacgc taataccgca

121 tacgacctac gggtgaaagc ggaggacctt cgggcttcgc gcgattgaat gagccgatgt

181 cggattagct agttggcggg gtaaaggccc accaaggcga cgatccgtag ctggtctgag

241 aggatgatca gccacactgg aactgagaca cggtccagac tcctacggga ggcagcagtg

301 gggaatattg gacaatgggc gcaagcctga tccagccatg ccgcgtgggt gaagaaggcc

361 ttcgggttgt aaagcccttt tgttgggaaa gaaaagcagt cggttaatac ccgattgttc

421 tgacggtacc caaagaataa gcaccggcta acttcgtgcc agcagccgcg gtaatacgaa

481 gggtgcaagc gttactcgga attactgggc gtaaagcgtg cgtaggtggt ggtttaagtc

541 tgttgtgaaa gccctgggct caacctggga attgcagtgg atactgggtc actagagtgt

601 ggtagagggt agcggaattc ccggtgtagc agtgaaatgc gtagagatcg ggaggaacat

661 ccgtggcgaa ggcggctacc tggaccaaca ctgacactga ggcacgaaag cgtggggagc

721 aaacaggatt agataccctg gtagtccacg ccctaaacga tgcgaactgg atgttgggtg

781 caatttggca cgcagtatcg aagctaacgc gttaagttcg ccgcctgggg agtacggtcg

841 caagactgaa actcaaagga attgacgggg gcccgcacaa gcggtggagt atgtggttta

901 attcgatgca acgcgaagaa ccttacctgg tcttgacatc cacggaactt tccagagatg

961 gattggtgcc ttcgggaacc gtgagacagg tgctgcatgg ctgtcgtcag ctcgtgtcgt

1021 gagatgttgg gttaagtccc gcaacgagcg caacccttgt ccttagttgc cagcacgtaa

1081 tggtgggaac tctaaggaga ccgccggtga caaaccggag gaaggtgggg atgacgtcaa

1141 gtcatcatgg cccttacgac cagggctaca cacgtactac aatggtaggg acagagggct

1201 gcaaacccgc gagggtaagc caatcccaga aaccctatct cagtccggat tggagtctgc

1261 aactcgactc catgaagtcg gaatcgctag taatcgcaga tcagcattgc tgcggtgaat

1321 acgttcccgg gccttgtaca caccgcccgt cacaccatgg gagtttgttg caccagaagc

1381 aggtagctta accttcggga gggcgc

//

LOCUS PQ722042 1379 bp DNA linear BCT 09-DEC-2024

DEFINITION Serratia sp. (in: enterobacteria) strain S-11 16S ribosomal RNA

gene, partial sequence.

ACCESSION PQ722042

VERSION PQ722042

KEYWORDS .

SOURCE Serratia sp. (in: enterobacteria)

ORGANISM Serratia sp. (in: enterobacteria)

Bacteria; Pseudomonadati; Pseudomonadota; Gammaproteobacteria;

Enterobacterales; Yersiniaceae; Serratia.

REFERENCE 1 (bases 1 to 1379)

AUTHORS Xun,L.

TITLE Direct Submission

JOURNAL Submitted (09-DEC-2024) College of Animal Science and Technology,

Yunnan Agricultural University, Fengyuan Road No. 452, Kunming,

Yunnan 650201, China

COMMENT ##Assembly-Data-START##

Sequencing Technology :: Sanger dideoxy sequencing

##Assembly-Data-END##

FEATURES Location/Qualifiers

source 1..1379

/organism="Serratia sp. (in: enterobacteria)"

/mol_type="genomic DNA"

/strain="S-11"

/db_xref="taxon:616"

/geo_loc_name="China: Yunnan, Tengchong County"

/collection_date="16-Jun-2024"

rRNA <1..>1379

/product="16S ribosomal RNA"

ORIGIN

1 gcggtagcac aggagagctt gctctctggg tgacgagcgg cggacgggtg agtaatgtct

61 gggaaactgc ctgatggagg gggataacta ctggaaacgg tagctaatac cgcataacgt

121 cttcggacca aagtggggga ccttcgggcc tcacgccatc agatgtgccc agatgggatt

181 agctagtagg tggggtaatg gctcacctag gcgacgatcc ctagctggtc tgagaggatg

241 accagccaca ctggaactga gacacggtcc agactcctac gggaggcagc agtggggaat

301 attgcacaat gggcgcaagc ctgatgcagc catgccgcgt gtgtgaagaa ggccttcggg

361 ttgtaaagca ctttcagcga ggaggaaggg tacagtgtta atagcacagt tcattgacgt

421 tactcgcaga agaagcaccg gctaactccg tgccagcagc cgcggtaata cggagggtgc

481 aagcgttaat cggaattact gggcgtaaag cgcacgcagg cggtttgtta agtcagatgt

541 gaaatccccg cgcttaacgt gggaactgca tttgaaactg gcaagctaga gtcttgtaga

601 ggggggtaga attccaggtg tagcggtgaa atgcgtagag atctggagga ataccggtgg

661 cgaaggcggc cccctggaca aagactgacg ctcaggtgcg aaagcgtggg gagcaaacag

721 gattagatac cctggtagtc cacgctgtaa acgatgtcga cttggaggtt gtgcccttga

781 ggcgtggctt ccggagctaa cgcgttaagt cgaccgcctg gggagtacgg ccgcaaggtt

841 aaaactcaaa tgaattgacg ggggcccgca caagcggtgg agcatgtggt ttaattcgat

901 gcaacgcgaa gaaccttacc tactcttgac atccagagaa ttcgctagag atagcttagt

961 gccttcggga actctgagac aggtgctgca tggctgtcgt cagctcgtgt tgtgaaatgt

1021 tgggttaagt cccgcaacga gcgcaaccct tatcctttgt tgccagcacg taatggtggg

1081 aactcaaagg agactgccgg tgataaaccg gaggaaggtg gggatgacgt caagtcatca

1141 tggcccttac gagtagggct acacacgtgc tacaatggcg tatacaaaga gaagcgaact

1201 cgcgagagca agcggacctc ataaagtacg tcgtagtccg gatcggagtc tgcaactcga

1261 ctccgtgaag tcggaatcgc tagtaatcgt agatcagaat gctacggtga atacgttccc

1321 gggccttgta cacaccgccc gtcacaccat gggagtgggt tgcaaaagaa gtaggtagc

//

LOCUS PQ722043 1374 bp DNA linear BCT 09-DEC-2024

DEFINITION Pseudomonas palleroniana strain S-12 16S ribosomal RNA gene,

partial sequence.

ACCESSION PQ722043

VERSION PQ722043

KEYWORDS .

SOURCE Pseudomonas palleroniana

ORGANISM Pseudomonas palleroniana

Bacteria; Pseudomonadati; Pseudomonadota; Gammaproteobacteria;

Pseudomonadales; Pseudomonadaceae; Pseudomonas.

REFERENCE 1 (bases 1 to 1374)

AUTHORS Xun,L.

TITLE Direct Submission

JOURNAL Submitted (09-DEC-2024) College of Animal Science and Technology,

Yunnan Agricultural University, Fengyuan Road No. 452, Kunming,

Yunnan 650201, China

COMMENT ##Assembly-Data-START##

Sequencing Technology :: Sanger dideoxy sequencing

##Assembly-Data-END##

FEATURES Location/Qualifiers

source 1..1374

/organism="Pseudomonas palleroniana"

/mol_type="genomic DNA"

/strain="S-12"

/db_xref="taxon:191390"

/geo_loc_name="China: Yunnan, Tengchong County"

/collection_date="16-Jun-2024"

rRNA <1..>1374

/product="16S ribosomal RNA"

ORIGIN

1 agagagaagc ttgcttctct tgagagcggc ggacgggtga gtaatgccta ggaatctgcc

61 tggtagtggg ggataacgtt cggaaacgga cgctaatacc gcatacgtcc tacgggagaa

121 agcaggggac cttcgggcct tgcgctatca gatgagccta ggtcggatta gctagttggt

181 gaggtaatgg ctcaccaagg cgacgatccg taactggtct gagaggatga tcagtcacac

241 tggaactgag acacggtcca gactcctacg ggaggcagca gtggggaata ttggacaatg

301 ggcgaaagcc tgatccagcc atgccgcgtg tgtgaagaag gtcttcggat tgtaaagcac

361 tttaagttgg gaggaagggc agttgcctaa tacgtaactg ttttgacgtt accgacagaa

421 taagcaccgg ctaactctgt gccagcagcc gcggtaatac agagggtgca agcgttaatc

481 ggaattactg ggcgtaaagc gcgcgtaggt ggtttgttaa gttggatgtg aaatccccgg

541 gctcaacctg ggaactgcat tcaaaactga ctgactagag tatggtagag ggtggtggaa

601 tttcctgtgt agcggtgaaa tgcgtagata taggaaggaa caccagtggc gaaggcgacc

661 acctggactg atactgacac tgaggtgcga aagcgtgggg agcaaacagg attagatacc

721 ctggtagtcc acgccgtaaa cgatgtcaac tagccgttgg aagccttgag cttttagtgg

781 cgcagctaac gcattaagtt gaccgcctgg ggagtacggc cgcaaggtta aaactcaaat

841 gaattgacgg gggcccgcac aagcggtgga gcatgtggtt taattcgaag caacgcgaag

901 aaccttacca ggccttgaca tccaatgaac tttccagaga tggattggtg ccttcgggaa

961 cattgagaca ggtgctgcat ggctgtcgtc agctcgtgtc gtgagatgtt gggttaagtc

1021 ccgtaacgag cgcaaccctt gtccttagtt accagcacgt tatggtgggc actctaagga

1081 gactgccggt gacaaaccgg aggaaggtgg ggatgacgtc aagtcatcat ggcccttacg

1141 gcctgggcta cacacgtgct acaatggtcg gtacaaaggg ttgccaagcc gcgaggtgga

1201 gctaatccca taaaaccgat cgtagtccgg atcgcagtct gcaactcgac tgcgtgaagt

1261 cggaatcgct agtaatcgcg aatcagaatg tcgcggtgaa tacgttcccg ggccttgtac

1321 acaccgcccg tcacaccatg ggagtgggtt gcaccagaag tagctagtct aacc

//

LOCUS PQ722044 1404 bp DNA linear BCT 09-DEC-2024

DEFINITION Xanthomonas arboricola pv. pruni strain S-13 16S ribosomal RNA

gene, partial sequence.

ACCESSION PQ722044

VERSION PQ722044

KEYWORDS .

SOURCE Xanthomonas arboricola pv. pruni

ORGANISM Xanthomonas arboricola pv. pruni

Bacteria; Pseudomonadati; Pseudomonadota; Gammaproteobacteria;

Lysobacterales; Lysobacteraceae; Xanthomonas.

REFERENCE 1 (bases 1 to 1404)

AUTHORS Xun,L.

TITLE Direct Submission

JOURNAL Submitted (09-DEC-2024) College of Animal Science and Technology,

Yunnan Agricultural University, Fengyuan Road No. 452, Kunming,

Yunnan 650201, China

COMMENT ##Assembly-Data-START##

Sequencing Technology :: Sanger dideoxy sequencing

##Assembly-Data-END##

FEATURES Location/Qualifiers

source 1..1404

/organism="Xanthomonas arboricola pv. pruni"

/mol_type="genomic DNA"

/strain="S-13"

/db_xref="taxon:69929"

/geo_loc_name="China: Yunnan, Tengchong County"

/collection_date="16-Jun-2024"

/note="pathovar: pruni"

rRNA <1..>1404

/product="16S ribosomal RNA"

ORIGIN

1 tcgaacggca gccagtaaga gcttgctctt atgggtggcg agtggcggac gggtgaggaa

61 tacatcggaa tctactcttt cgtgggggat aacgtaggga aacttacgct aataccgcat

121 acgacctacg ggtgaaagcg gaggaccttc gggcttcgcg cgattgaatg agccgatgtc

181 ggattagcta gttggcgggg taaaggccca ccaaggcgac gatccgtagc tggtctgaga

241 ggatgatcag ccacactgga actgagacac ggtccagact cctacgggag gcagcagtgg

301 ggaatattgg acaatgggcg caagcctgat ccagccatgc cgcgtgggtg aagaaggcct

361 tcgggttgta aagccctttt gttgggaaag aaaagcagtc ggttaatacc cgattgttct

421 gacggtaccc aaagaataag caccggctaa cttcgtgcca gcagccgcgg taatacgaag

481 ggtgcaagcg ttactcggaa ttactgggcg taaagcgtgc gtaggtggtg gtttaagtct

541 gttgtgaaag ccctgggctc aacctgggaa ttgcagtgga tactgggtca ctagagtgtg

601 gtagagggta gcggaattcc cggtgtagca gtgaaatgcg tagagatcgg gaggaacatc

661 cgtggcgaag gcggctacct ggaccaacac tgacactgag gcacgaaagc gtggggagca

721 aacaggatta gataccctgg tagtccacgc cctaaacgat gcgaactgga tgttgggtgc

781 aatttggcac gcagtatcga agctaacgcg ttaagttcgc cgcctgggga gtacggtcgc

841 aagactgaaa ctcaaaggaa ttgacggggg cccgcacaag cggtggagta tgtggtttaa

901 ttcgatgcaa cgcgaagaac cttacctggt cttgacatcc acggaacttt ccagagatgg

961 attggtgcct tcgggaaccg tgagacaggt gctgcatggc tgtcgtcagc tcgtgtcgtg

1021 agatgttggg ttaagtcccg caacgagcgc aacccttgtc cttagttgcc agcacgtaat

1081 ggtgggaact ctaaggagac cgccggtgac aaaccggagg aaggtgggga tgacgtcaag

1141 tcatcatggc ccttacgacc agggctacac acgtactaca atggtaggga cagagggctg

1201 caaacccgcg agggtaagcc aatcccagaa accctatctc agtccggatt ggagtctgca

1261 actcgactcc atgaagtcgg aatcgctagt aatcgcagat cagcattgct gcggtgaata

1321 cgttcccggg ccttgtacac accgcccgtc acaccatggg agtttgttgc accagaagca

1381 ggtagcttaa ccttcgggag ggcg

//

LOCUS PQ721930 521 bp DNA linear PLN 09-DEC-2024

DEFINITION Metschnikowia reukaufii strain Y-1 large subunit ribosomal RNA

gene, partial sequence.

ACCESSION PQ721930

VERSION PQ721930

KEYWORDS .

SOURCE Metschnikowia reukaufii

ORGANISM Metschnikowia reukaufii

Eukaryota; Fungi; Dikarya; Ascomycota; Saccharomycotina;

Pichiomycetes; Metschnikowiaceae; Metschnikowia.

REFERENCE 1 (bases 1 to 521)

AUTHORS Xun,L.

TITLE Direct Submission

JOURNAL Submitted (09-DEC-2024) College of Animal Science and Technology,

Yunnan Agricultural University, Fengyuan Road No. 452, Kunming,

Yunnan 650201, China

COMMENT ##Assembly-Data-START##

Sequencing Technology :: Sanger dideoxy sequencing

##Assembly-Data-END##

FEATURES Location/Qualifiers

source 1..521

/organism="Metschnikowia reukaufii"

/mol_type="genomic DNA"

/strain="Y-1"

/db_xref="taxon:27327"

/geo_loc_name="China: Yunnan, Tengchong County"

/collection_date="16-Jun-2024"

rRNA <1..>521

/product="large subunit ribosomal RNA"

ORIGIN

1 gaggaaaaga aaccaacagg gattgcctca gtaacggcga gtgaagcggc aaaagctcaa

61 atttgaaatc ccccgggaat tgtaatttga aggacctatt aaaaccctga gagagcccaa

121 agtccattgg aaaatggcgc catagaaggt gatagccctg tgtggacctc tctcagcata

181 ctttttaggc ccaaagagtc gagttgtttg ggaatgcagc tctaagtggg tggtaaattc

241 catctaaagc taaatattgg cgagagaccg atagcgaaca agtacagtga tggaaagatg

301 aaaagcactt tgaaaagaga gtgaaaaagt acgtgaaatt gttgaaaggg aagggcttgc

361 aagcagacac aacctcggtt gggccagcat cggggcgggg ggaaacaaaa aaggtgtgga

421 atgtggctct tacgagtgtt atagccccac ccaatatttc catcccatcc cgaggcctgc

481 gattcttcaa ggatgctggc gtaatggttg caagtcgccc g

//

LOCUS PQ721931 524 bp DNA linear PLN 09-DEC-2024

DEFINITION Metschnikowia reukaufii strain Y-2 large subunit ribosomal RNA

gene, partial sequence.

ACCESSION PQ721931

VERSION PQ721931

KEYWORDS .

SOURCE Metschnikowia reukaufii

ORGANISM Metschnikowia reukaufii

Eukaryota; Fungi; Dikarya; Ascomycota; Saccharomycotina;

Pichiomycetes; Metschnikowiaceae; Metschnikowia.

REFERENCE 1 (bases 1 to 524)

AUTHORS Xun,L.

TITLE Direct Submission

JOURNAL Submitted (09-DEC-2024) College of Animal Science and Technology,

Yunnan Agricultural University, Fengyuan Road No. 452, Kunming,

Yunnan 650201, China

COMMENT ##Assembly-Data-START##

Sequencing Technology :: Sanger dideoxy sequencing

##Assembly-Data-END##

FEATURES Location/Qualifiers

source 1..524

/organism="Metschnikowia reukaufii"

/mol_type="genomic DNA"

/strain="Y-2"

/db_xref="taxon:27327"

/geo_loc_name="China: Yunnan, Tengchong County"

/collection_date="16-Jun-2024"

rRNA <1..>524

/product="large subunit ribosomal RNA"

ORIGIN

1 ggaggaaaag aaaccaacag ggattgcctc agtaacggcg agtgaagcgg caaaagctca

61 aatttgaaat cccccgggaa ttgtaatttg aaggacctat taaaaccctg agagagccca

121 aagtccattg gaaaatggcg ccatagaagg tgatagccct gtgtggacct ctctcagtat

181 actttttagg cccaaagagt cgagttgttt gggaatgcag ctctaagtgg gtggtaaatt

241 ccatctaaag ctaaatattg gcgagagacc gatagcgaac aagtacagtg atggaaagat

301 gaaaagcact ttgaaaagag agtgaaaaag tacgtgaaat tgttgaaagg gaagggcttg

361 caagcagaca caacctcggt tgggccagca tcggggcggg gggaaacaaa aaaggtgtgg

421 aatgtggctc ttacgagtgt tatagcccca cccaatattt ccatcccatc ccgaggcctg

481 cgattcttca aggatgctgg cgtaatggtt gcaagtcgcc cgtc

//
